# Supplementary material for: CUL4A-DDB1-DCAF10 is an N-recognin for N-terminally acetylated Src kinases
Source: Nat Commun. 2026 Jan 3;17:132. doi: 10.1038/s41467-025-68074-9 (PMC12775124; doi:10.1038/s41467-025-68074-9)
Supplement: Supplementary file 1 — Supplementary Information [file 41467_2025_68074_MOESM1_ESM.pdf]

# Supplementary Information

# CUL4A-DDB1-DCAF10 is an N-recognin for N-terminally acetylated Src kinases

Nora Kremer[1,2,§], Franziska Mueller[3,§], Hang Nguyen[1,2], Louisa Schulz[1,2], Tanja Popp[1], Elena Artes[1,2], Julian Wolters[1], Michael Renner[1,2], Ingrid Vetter[3], Stefano Maffini[3], Maria S. Robles[2], Andrea Musacchio[3,4], Tanja Bange[1,2,*]

1 Department of Medicine II, LMU University Hospital, Munich, Germany

2 Institute of Medical Psychology and Biomedical Center (BMC), Faculty of Medicine, LMU Munich, 80336 Munich, Germany

3 Department of Mechanistic Cell Biology, Max Planck Institute of Molecular Physiology, 44227 Dortmund, Germany

4 Centre for Medical Biotechnology, Faculty of Biology, University of Duisburg-Essen, 45141 Essen, Germany

[§] These authors contributed equally.

[*] Address correspondence to:

T.B.: tanja.bange@med.uni-muenchen.de, +49 89 4400 76180, ORCID 0000-0002-9680-8586

**Keywords:** N-degron pathway, Src-family kinases, Nt-acetylation, N-myristoylation, ubiquitination, CUL4A-DDB1-DCAF10

# Table of contents

**Supplementary Figures and Legends**

- Supplementary Fig. 1
- Supplementary Fig. 2
- Supplementary Fig. 3
- Supplementary Fig. 4
- Supplementary Fig. 5
- Supplementary Fig. 6
- Supplementary Fig. 7
- Supplementary Fig. 8
- Supplementary Fig. 9

**Supplementary Methods**

**Supplementary Material Tables**

- Supplementary Material Table 1. Peptides
- Supplementary Material Table 2. Cell lines
- Supplementary Material Table 3. siRNAs
- Supplementary Material Table 4. Oligonucleotides and primers
- Supplementary Material Table 5. Plasmids
- Supplementary Material Table 6. Antibodies
- Supplementary Material Table 7. Antibiotics used in bacterial and cell culture
- Supplementary Material Table 8. Reagents and Kits
- Supplementary Material Table 9. List of enzymes
- Supplementary Material Table 10. Software and Algorithms

**Supplementary References**

**Supplementary Data Tables (Mass spectrometry data provided as Excel sheets):**

- Supplementary Data Table 1. MS data volcano plots to **Figure 1**
- Supplementary Data Table 2. MS data volcano plots to **Supplementary Fig. 1**
- Supplementary Data Table 3. MS data volcano plots to **Figure 2**
- Supplementary Data Table 4. MS data DCAF10 fractionation to **Figure 4**
- Supplementary Data Table 5. MS data ZYG11B fractionation to **Figure 4**
- Supplementary Data Table 6. MS data Lyn parental and KOs to **Supplementary Fig.**

**7**

- Supplementary Data Table 7. MS data Ac-Ala and P volcano plots to **Supplementary Fig. 7**
- Supplementary Data Table 8. MS data IP Lyn-GFP volcano plots to **Fig. 6**

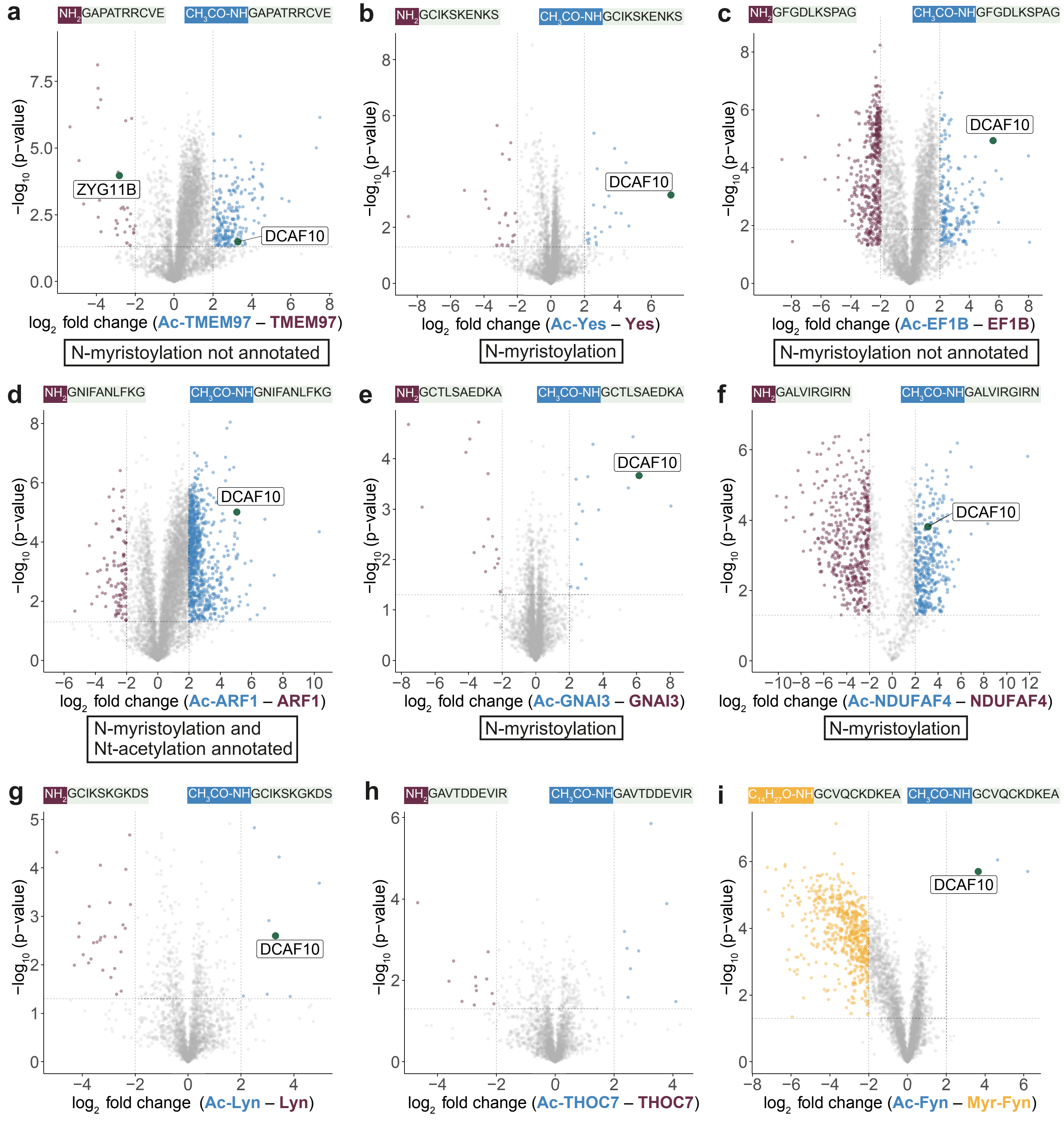


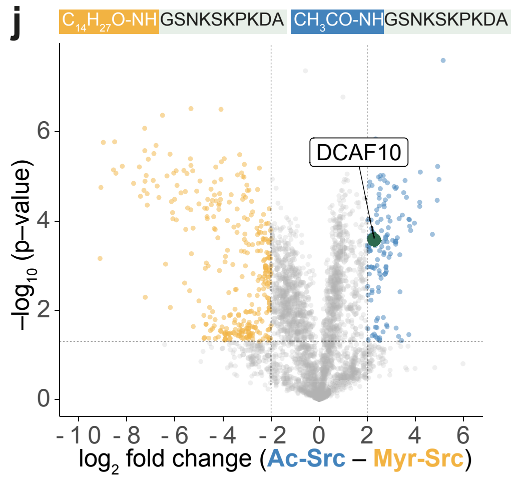


**k**

Raw $\log_2$ LFQ intensities of DCAF10 of Ac versus Myr peptides:

| Gene Name | Ac_1 | Ac_2 | Ac_3 | Myr_1 | Myr_2 | Myr_3 |
|---|---|---|---|---|---|---|
| Lyn | 24.95 | 24.89 | 24.77 | 20.72 | 21.14 | 20.48 |
| Fyn | 28.18 | 28.13 | 28.33 | 22.33 | 22.32 | 22.49 |
| Src | 19.41 | 19.36 | 19.28 | NaN | NaN | NaN |

Supplementary Figure 1 Kremer et al.

## Supplementary Figures and Legends

**Supplementary Fig. 1. DCAF10 binds acetylated N-terminal glycine residues.**

**a-f**) Volcano plots comparing binding partners of free (Nt-free) and acetylated (Nt-Ac) N-termini of peptides derived from the indicated proteins (aa 1–10 + KKK-biotin; Supplementary Table S1). The analyzed peptides are from (**a**) TMEM97, (**b**) Yes, (**c**) EF1B, (**d**) ARF1, (**e**) GNAI3, (**f**) NDUFAF4. The -log10 adjusted p-value (two-sided Student's t-test with permutation-based multiple-testing correction; y-axis) is plotted against the log2 fold change (x-axis). Threshold for significance: -log10 p-value ≥ 1.3 (p-value ≤ 0.05), log2 fold change ≤ -2 or ≥ 2. DCAF10 and ZYG11B are marked in green, significant Nt-free binders in purple, and significant Nt-Ac binders in blue ($n$ = 3 independent biological replicates). **g-h**) Volcano plots as in (**a-f**) using homogenized liver tissue from mice. (**g**) Lyn, (**h**) THOC7. **i-j**) Volcano plots as in (**a-f**) comparing binding partners of Nt-Ac and Nt-Myr (**i**) Fyn and (**j**) Src. In addition to (**a-f**), significant Nt-Myr binders are marked in yellow. **k**) Overview table showing raw log2 LFQ intensities before data imputation comparing the abundance of DCAF10 in Nt-Ac and Nt-Myr forms of Lyn, Fyn, and Src.

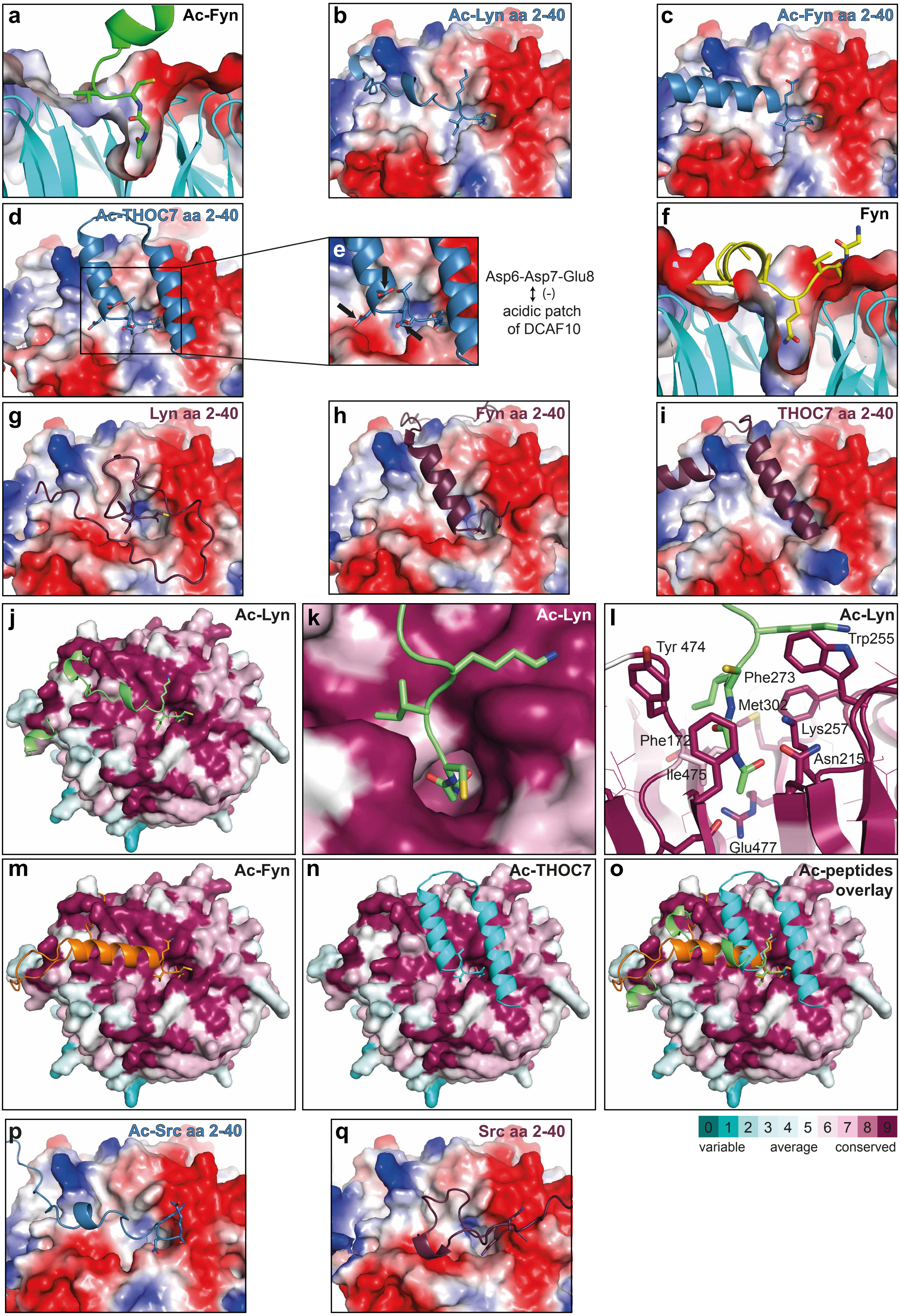


Supplementary Figure 2 Kremer et al.

**Supplementary Fig. 2. AF3 predictions.**

**a-f**) Cross-sections of the DCAF10 β-propeller (aa 120–559, UniprotID: Q5QP82), highlighting the deep tunnel bound to Nt-Fyn peptides. Shown are **a**) Nt-Ac Fyn (green), **f**) Nt-free Fyn (yellow). **b-e**) Top views of the DCAF10 β-propeller bound to the first 39 aa (aa 2–40) of various proteins. Nt-Ac aa 2–40: **b**) Nt-Ac Lyn, **c**) Nt-Ac Fyn, **d**) Nt-Ac THOC7 (black box indicates region shown in (**e**)). **e**) Close-up view of (**d**): interaction of Asp6-Asp7-Glu8 (shown as sticks) of THOC7 with the acidic DCAF10 surface. Nt-free aa 2–40: **g**) Nt-free Lyn, **h**) Nt-free Fyn, **i**) Nt-free THOC7. DCAF10's electrostatic surface potential is displayed (red = negative, blue = positive, white = neutral or hydrophobic), as calculated by PyMOL. Acetylated fragments insert deeply into the central DCAF10 tunnel, whereas non-acetylated peptides are consistently excluded. **j-o**) Conservation analysis of the DCAF10 β-propeller. Color code: cyan: variable, white: moderate conservation, purple: highly conserved. **j**) Full β-propeller view with Nt-Ac Lyn, **k**) Close-up of the tunnel entrance with Nt-Ac Lyn; **l**) Interacting residues in DCAF10. **m-o**) Additional Nt-Ac fragments aa 2–40: **m**) Nt-Ac Fyn (orange), **n**) Nt-Ac THOC7 (cyan), **o**) overlay of Nt-Ac Lyn, Fyn, and THOC7 models. **p-q)** Cross-sections of the DCAF10 β-propeller with (**p**) Nt-Ac Src; (**q**) Nt-free Src. Panels (**p–q**) use the same color code as panels (**a–f**).

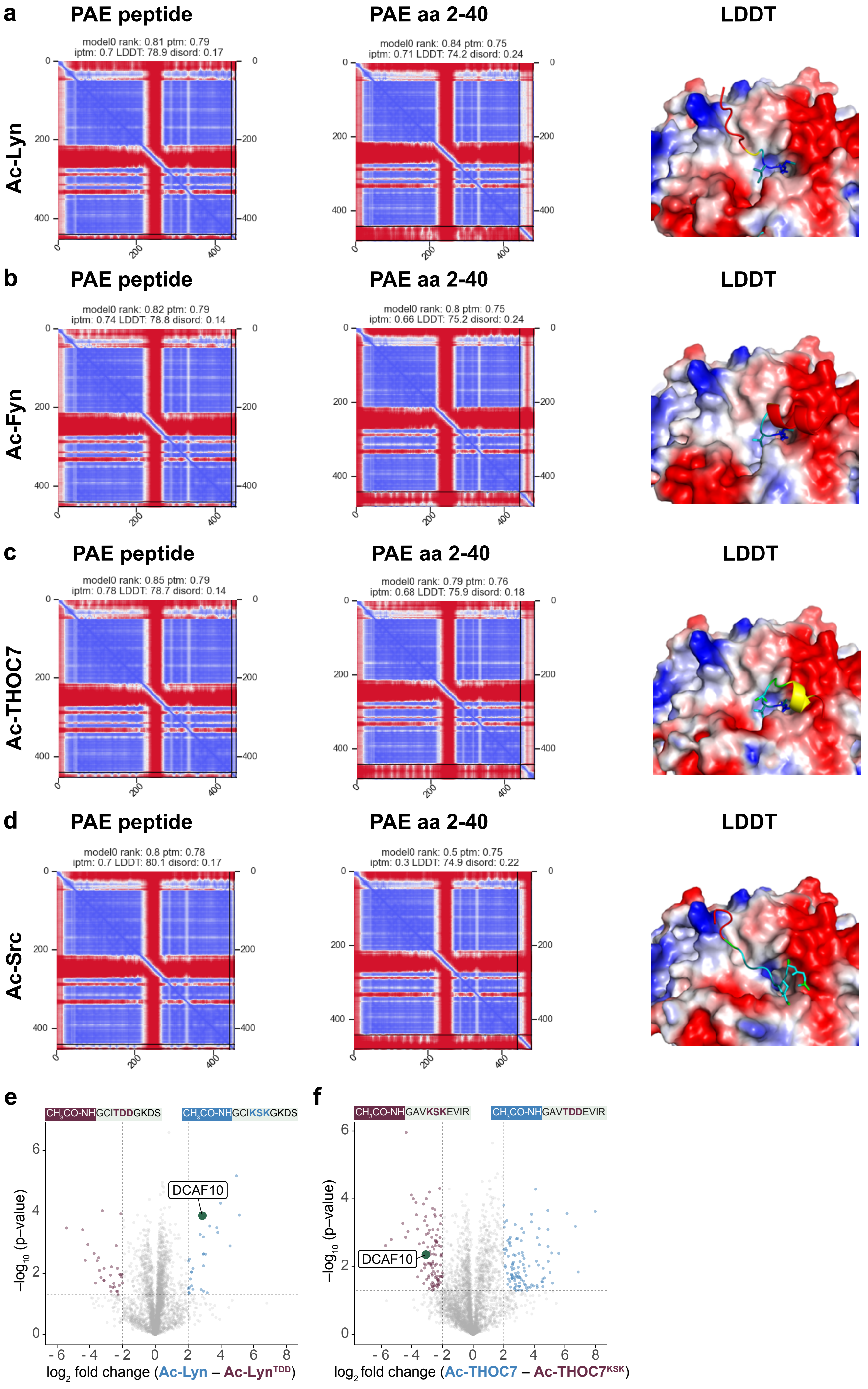


Supplementary Figure 3 Kremer et al.

**Supplementary Fig. 3. PAE and LLDT maps of AF3 predictions, and volcano plots of N-terminal peptide variants.**

**a-d**) PAE (Predicted Aligned Error) plots for the best models and LDDT (Local Distance Difference Test) maps of AF3 models for DCAF10 binding to Ac-peptides and fragments (aa 2–40). Left: PAE plots of Nt-Ac peptides (aa 1–10 + KKK-biotin). Middle: PAE plots of aa 2–40. Right: LDDT maps of Nt-Ac peptides. Analyzed peptides (**a**) Nt-Ac Lyn, (**b**) Nt-Ac Fyn, (**c**) Nt-Ac THOC7, (**d**) Nt-Ac Src. PAE plots are color-coded: blue (highly significant scores), red (insignificant). LDDT maps are color-coded: blue (very high confidence, LDDT > 90), cyan (high confidence, LDDT 70–90), yellow (medium confidence, LDDT 50–70), orange (low confidence, LDDT 30–50), and red (very low confidence, LDDT < 30). The DCAF10 surface is colored by electrostatic potential as in **Supplementary Fig. 2**. **a-f**). **e-f**) Volcano plots comparing binding partners of (**e**) Ac-Lyn vs. Ac-Lyn$^{TDD}$ and (**f**) Ac-THOC7 vs. Ac-THOC7$^{KSK}$. The -log10 adjusted p-value (two-sided Student's t-test with permutation-based multiple-testing correction; y-axis) is plotted against the log2 fold change (x-axis). Threshold for significance: -log10 p-value ≥ 1.3 (p-value ≤ 0.05), log2 fold change ≤ -2 or ≥ 2. DCAF10 is marked in green, significant binding partners in purple and blue (*n* = 3 independent biological replicates).

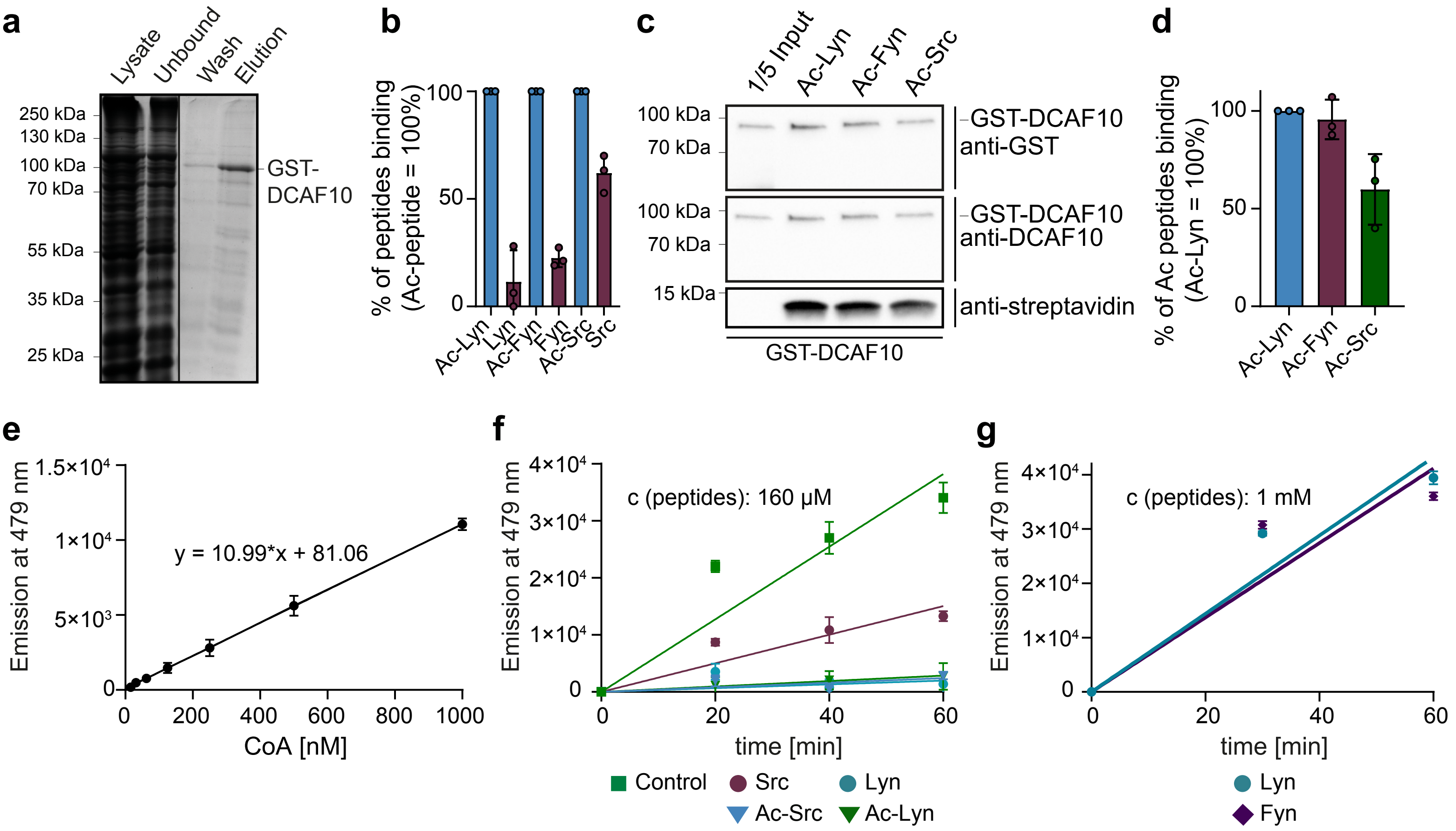


Supplementary Figure 4 Kremer et al.

**Supplementary Fig. 4. *In vitro* binding of DCAF10 to Nt-Ac peptides and *in vitro* Nt-acetylation assay setup.**

**a**) Coomassie-stained SDS-PAGE gel showing GST-DCAF10 purification. **b**) WB quantification of **Fig. 2m-p**. Mean ± s.d. with individual data points are shown (*n* = 3 independent binding assays). **c**) Peptide pull-downs with Ac-peptides of Lyn, Fyn and Src and GST-DCAF10 as bait. **d**) Quantification of WBs; Lyn binding set to 100%; mean ± s.d. with individual data points are shown (*n* = 3 independent binding assays). **e**) Fluorescent CPM signal as a function of CoA-SH concentration. The linear regression of this graph was used to calculate Nt-acetylation levels and Michaelis-Menten kinetics. **f-g**) Time-courses of *in vitro* Nt-acetylation assays for Lyn, Ac-Lyn, Src, Ac-Src, Fyn, and a control peptide. 160 µM peptides (**f**) or 1mM (**g**) were incubated with 20 nM NatA and 80 µM acetyl-CoA for 20, 40 and 60 min to assess the linear range of the reaction. Data represent the mean ± standard deviation (*n* = 4 independent enzyme assays).

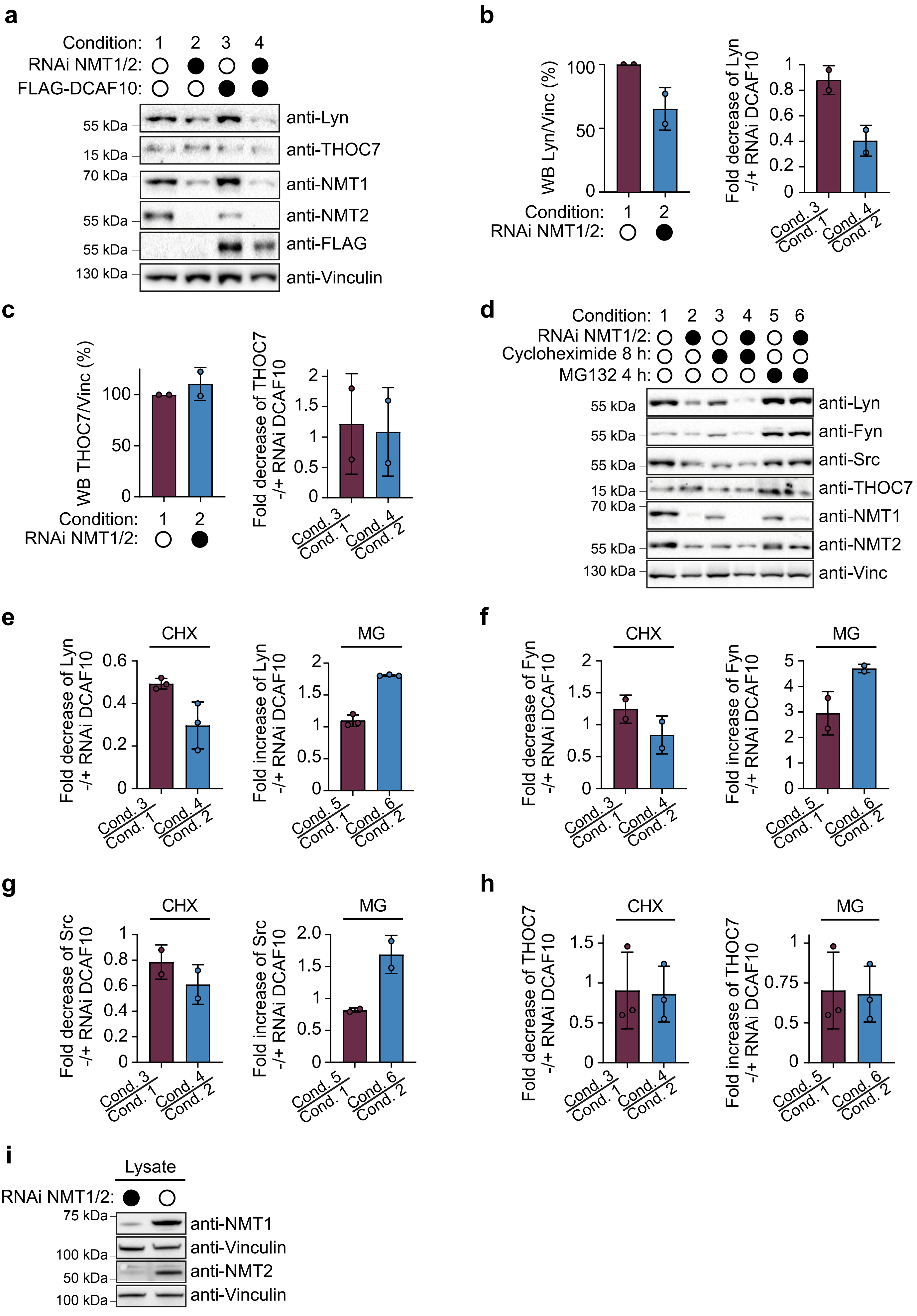


Supplementary Fig. 5 Kremer et al.

**Supplementary Fig. 5. NMT1/2 knockdown leads to an increased turnover of SFKs and overexpression of DCAF10 reduces SFK levels after NMT1/2 knockdown.** **a**) HeLa cells were treated with NMT1/2 siRNA for 72 h or left untreated in combination with FLAG-DCAF10 overexpression for 24 h. Representative WBs are shown. **b-c**) Left: WB quantifications with and without NMT1/2 siRNA of Lyn (**b**) and THOC7 (**c**) normalized to vinculin. Right: Fold change of Lyn (**b**) and THOC7 (**c**) after DCAF10 overexpression. Untreated: purple; with NMT1/2 RNAi: blue. Individual data points are indicated (*n* = 2 or 3 independent biological replicates). MG132 = proteasome inhibitor; CHX = cycloheximide translational inhibitor. **d**) HeLa cells were treated for 72 h with and without NMT1/2 siRNA and treated with cycloheximide or MG132 for the indicated periods of time. Representative WBs are shown. Filled circles indicate treatment; empty circles indicate no treatment. **e-h**) Fold-change analysis of protein expression levels under the indicated conditions. Left: fold change after 8 h cycloheximide treatment of (**e**) Lyn, (**f**) Fyn, (**g**) Src and (**h**) THOC7. Right: fold change after 4 h MG132 treatment of (**e**) Lyn, (**f**) Fyn, (**g**) Src and (**h**) THOC7. Untreated: purple; with NMT1/2 siRNA: blue (*n* = 3 independent biological replicates). **i**) WB of cellular lysates used to immunoprecipitate Lyn for *in vitro* ubiquitination assays in **Figure 5**. Source data are provided as a Source Data file.

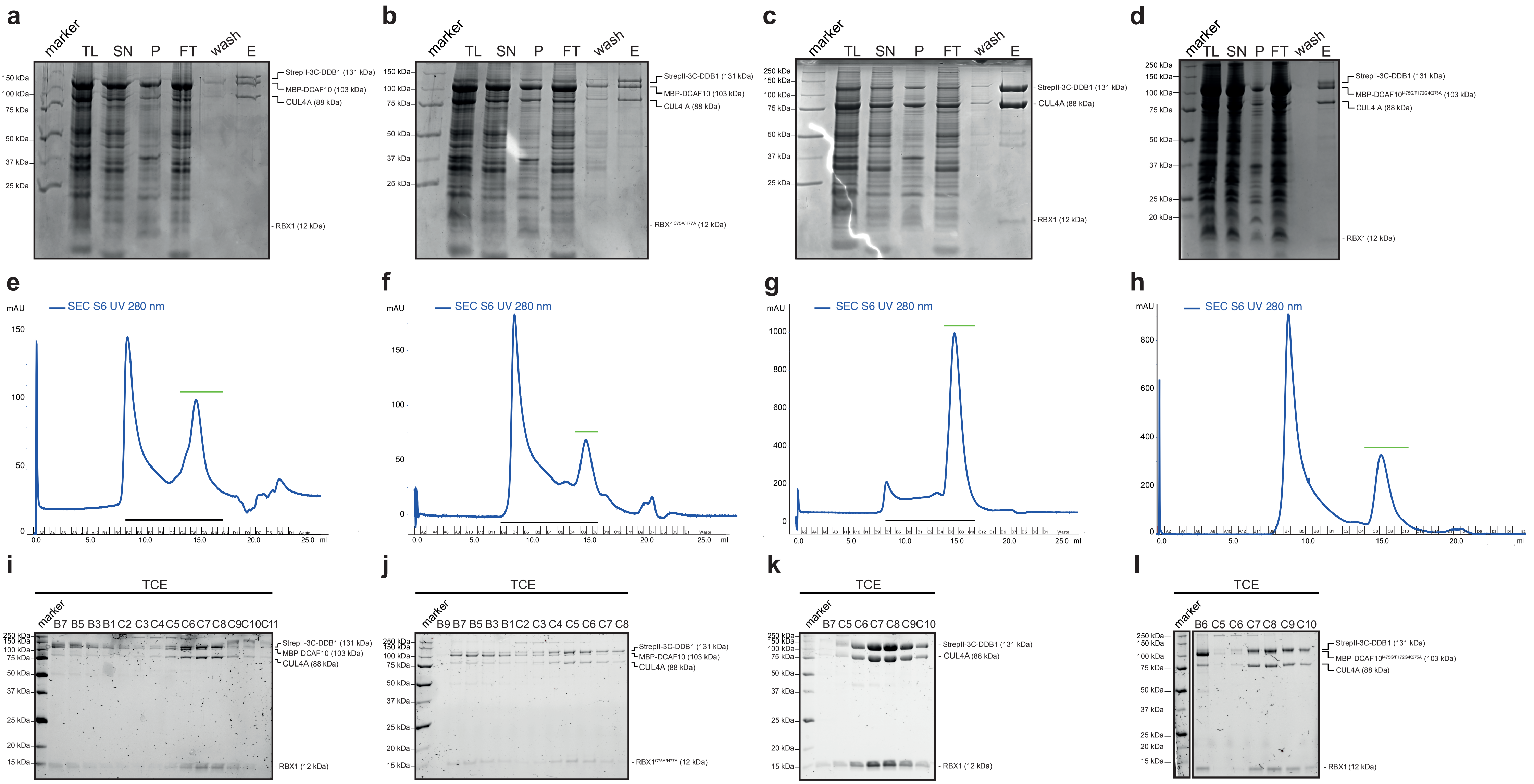


Supplementary Figure 6 Kremer et al.

**Supplementary Fig. 6. Purification of CUL4A, RBX1, StrepII-DDB1, 6xHIS-MBP-DCAF10 complexes.**

**a-d**) Affinity purifications of the E3 ligase full-length wild-type complex (StrepII-DDB1, CUL4A, RBX1, 6xHIS-MBP-DCAF10), RBX1$^{mut}$ complex (StrepII-DDB1, CUL4A, RBX1$^{C75A/H77A}$, 6xHIS-MBP-DCAF10), -DCAF10 complex (StrepII-DDB1, CUL4A, RBX1) and DCAF10$^{mut}$ complex (StrepII-DDB1, CUL4A$^{I475G/F172G/K257A}$, RBX1, 6xHISMBP-DCAF10) using Strep-Tactin®XT 4Flow® beads to purify the whole complex via the Strep-II tag fused to DDB1 from insect cells. Coomassie brilliant blue staining of SDS-PAGE gels are shown total lysate (TL), soluble supernatant (SN), pellet (P) and unbound flowthrough (FT) after incubation with beads, the wash of beads (wash), and the eluate (E). **e-h**) Strep-II tag purifications were run on a size-exclusion (SEC) column. Chromatograms of absorbance at A280 are shown. Black lines correspond to fractions visualized on SDS gels below (**i-h**), green lines to the pooled fractions taken for the in vitro ubiquitination assay. **i-h**) Fractions from (**e-h**) (black line) loaded on SDS gels and visualized via TCE (2,2,2-trichloroethanol) gels. Source data are provided as a Source Data file.

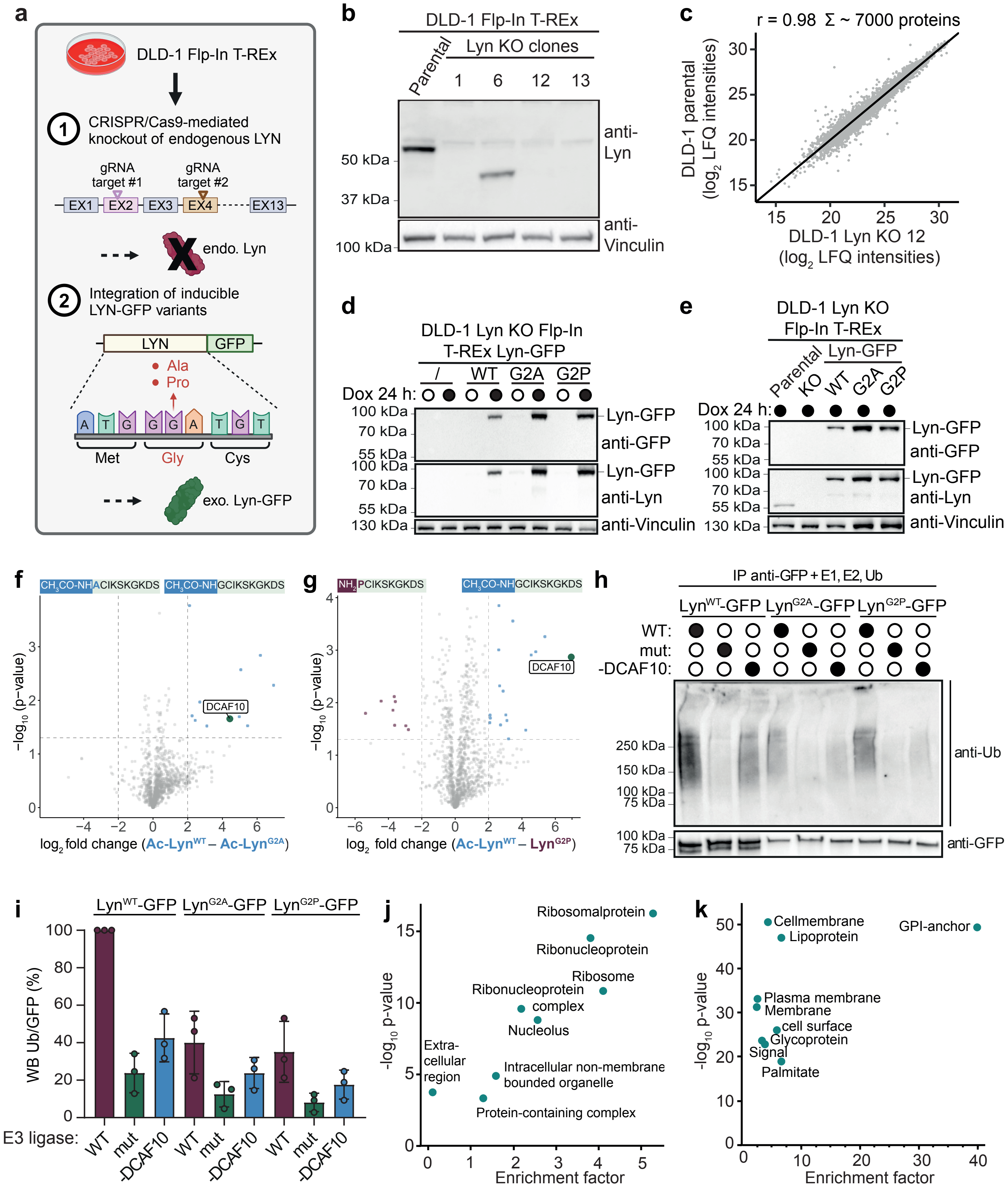


Supplementary Figure 7 Kremer et al.

**Supplementary Fig. 7. Generation of DLD-1 Lyn KO cell lines expressing doxycycline inducible Lyn-GFP variants (WT, G2A or G2P) and supporting experiments.**

**a**) Strategy for cell line generation: (1) Endogenous Lyn in parental DLD-1 Flp-In T-REx cells was knocked out (KO) using the CRISPR/Cas9. (2) Lyn-GFP WT and Nt-variants (G2A, G2P) were stably integrated into the Lyn KO genome. *Created in BioRender. Bange, T. (2025) https://BioRender.com/z58tr5a*. **b**) Validation of Lyn KO. WBs against Lyn and vinculin confirm disruption of endogenous Lyn in 75% of selected clones; clone 12 was used for stable integration. **c**) MS-based proteome comparison of parental and Lyn KO clone 12 cells. Log2 intensity values show comparable proteomes (r = 0.98; ~7000 quantified proteins). $N$ = 3 independent biological replicates **d**) Induction of Lyn-GFP variants with 100 ng/mL doxycycline (24 h) in Lyn KO cells. WBs confirm absence of endogenous Lyn and doxycycline-dependent expression of Lyn-GFP variants. **e**) Comparison of endogenous versus induced Lyn-GFP signals in parental and Lyn-GFP cell lines after 24 h doxycycline treatment. ($n$ = 3 independent biological replicates for **d-e**). **f-g**) Volcano plots comparing binding partners of mutant Nt-peptides of Lyn (aa 1–10 + KKK-biotin; **Supplementary Table 1**). **f**) Ac-Ala vs. AcGly, **g**) Pro vs. Ac-Gly. Volcano plots were generated using log2 fold changes (x-axis) and –log10 adjusted p-values (y-axis) from a two-sided Student's $t$-test with permutation-based multiple-testing correction (significance thresholds: –log10 $p \geq$ 1.3 and log2 fold change $\leq$ –2 or $\geq$ 2); DCAF10 marked in green, significant Nt-free binders in purple, Nt-Ac binders in blue ($n$ = 3 independent biological replicates). **h**) DLD-1 Lyn$^{WT}$-GFP, Lyn$^{G2A}$-GFP and Lyn$^{G2P}$-GFP cells were induced with doxycycline for 24 h. Lyn-GFP variants were immunoprecipitated using anti-GFP beads and subjected to *in vitro* ubiquitination assays with WT, RBX1$^{mut}$, and -DCAF10 complexes. **i**) Quantification of Ub signals normalized to GFP; mean ± s.d. with individual data points are shown ($n$ = 3 independent enzymatic assays). **j-k**) Fisher´s exact test of significant interaction partners of Lyn$^{WT}$-GFP IPs (**Fig. 6j-l)** (adjusted p-value

with Benjamini-Hochberg correction < 0.01). The -log10 adjusted p-value of the test (y-axis) is blotted against the enrichment factor (x-axis). (**j**) NMT1/2 RNAi treated lysates ± doxycycline (Dox); (**k**) control lysates ± doxycycline (Dox).

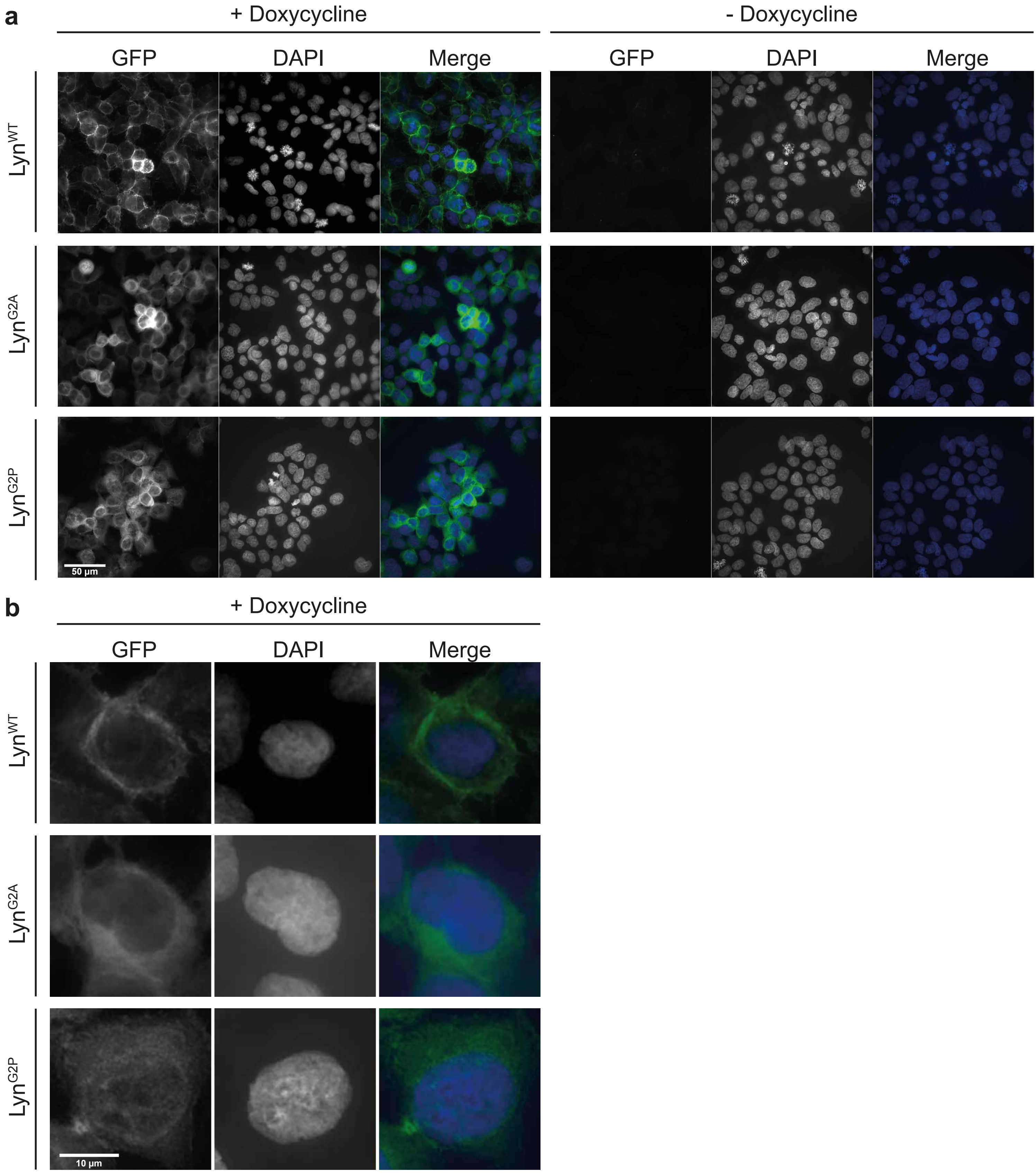


Supplementary Figure 8 Kremer et al.

**Supplementary Fig. 8. Fluorescence of DLD-1 Lyn KO Flp-In T-REx cells expressing $\text{Lyn}^{\text{WT}}$-GFP, $\text{Lyn}^{\text{G2A}}$-GFP or $\text{Lyn}^{\text{G2P}}$-GFP.**

**a)** Lyn (WT, G2A or G2P)-GFP expression was induced for 24 h with 100, 10 and 10 ng/mL doxycycline, respectively (left panel), or no doxycycline was added as control (right panel). GFP fluorescence and DAPI signals, used to visualize nuclei of fixed cells, were imaged with a 60x objective, confirming that all cells express Lyn-GFP upon doxycycline treatment. **b)** Cropped image of a representative cell for each Nt-variant from the images in **(a)**, showing Lyn-GFP localization.

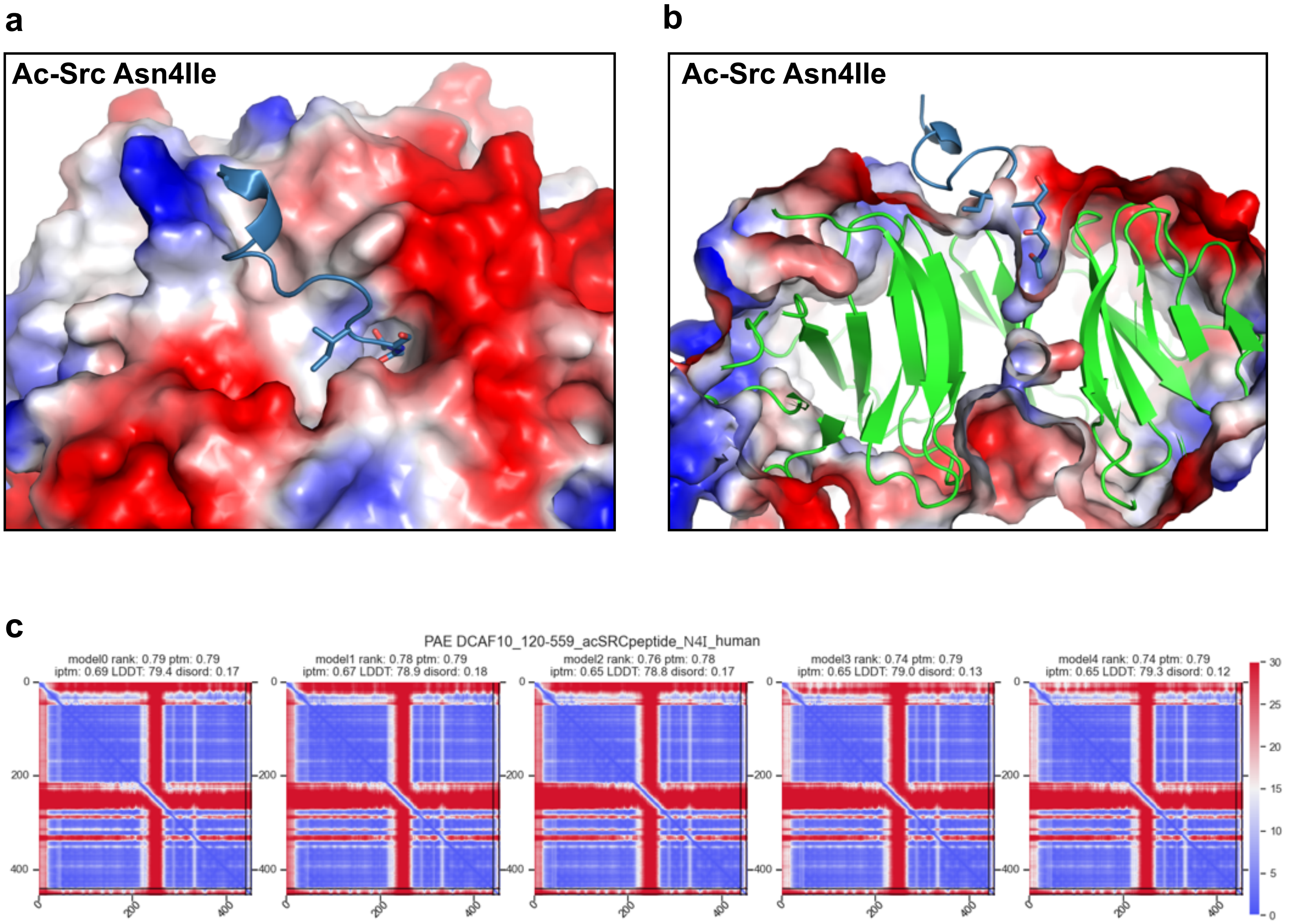


Supplementary Fig. 9 Kremer et. al.

**Supplementary Fig. 9. AF3 predictions of Src Asn4Ile mutant.**

**a)** Top view of DCAF10 (aa 120–559, UniprotID: Q5QP82) with Nt-Ac Src Asn4Ile (aa2-40). DCAF10's electrostatic surface potential is displayed (red = negative, blue = positive, white = neutral or hydrophobic), as calculated by PyMOL **b)** Cross section of DCAF10 (aa 120-559) with Src Asn4Ile (blue). **c)** PAE (Predicted Aligned Error) plots for the best AF3 model for DCAF10 binding to Nt-Ac Src Asn4Ile (aa 2–40).

## Supplementary Methods

### Peptide pull-downs

Peptide sequences are listed in **Supplementary Table 1**. Peptide pull-downs were performed as previously described[1]. Briefly, magnetic streptavidin beads (Dynabeads™ MyOne™ Streptavidin T1; Thermo Fisher Scientific, Waltham, MA, USA) were incubated with biotinylated peptides (4 µg of peptide/50 µL of bead slurry) for 30 min at room temperature (RT) in PBS. Beads were then washed three times with PBS. After washing, 1 mg of HeLa lysates or 2 mg of liver lysates was taken, and lysis buffer (75 mM HEPES pH 7.5, 150 mM KCl, 1.5 mM EGTA pH 8.0, 1.5 mM $MgCl_2$, 10% (v/v) glycerol, 0.075% NP-40, supplemented with 1mM DTT, PhosSTOP, and Protease Inhibitor Cocktail) was added to each sample. Lysates were incubated with streptavidin beads for 16 h at 4°C. HeLa pull-downs were washed twice with lysis buffer and twice with wash buffer (lysis buffer without glycerol, NP-40, DTT, PhosSTOP, and Protease Inhibitor Cocktail). Liver pulldowns were washed twice with cold low salt buffer (50 mM HEPES pH 7.5, 140 mM NaCl, 1% Triton X-100), once with cold high salt buffer (50 mM HEPES pH 7.5, 500 mM NaCl, 1% Triton X-100), and once with TBS. Organs were isolated from male C57BL/6N mice of 8–10 weeks of age that were maintained in individually ventilated cages under a 12-h light/dark cycle and were provided with sterile food and water under specific-pathogen-free conditions. All procedures were performed in accordance with European law regarding the protection of animal welfare and with approval by the local government authorities (animal facility registered for breeding and use of animals for scientific purpose KVR-I/221-TA166/22_03-06). Mouse liver tissue used in this study consisted of surplus material from animals euthanized for unrelated experiments in this laboratory. No animal procedures were performed specifically for this study, and no animals were handled by the authors. After withdrawing the supernatant, samples were directly digested on beads and processed for MS analysis. Samples were

reduced, alkylated, digested directly on beads with LysC/Trypsin (protein:enzyme ratio of 1:100), and desalted/concentrated on C18 reversed phase stage tips as previously described[24,56]. MS measurements settings are stated in **MS measurements and raw file processing**.

## Purification of GST-DCAF10

DCAF10 was amplified from pcDNA3.1+/C-(K)-DYK (GenScript; Piscataway, New Jersey, USA) and, via Gibson cloning, integrated into the first cassette of pGEX-6P-2rbs (GenBank accession code KM817768)[57]. BL21(DE3) cells (Thermo Fisher Scientific, Waltham, MA, USA) transformed with pGEX-6P-2rbs-DCAF10 were inoculated from a single colony picked from a Lysogeny Broth (LB) agar (LB medium with 1.5% (w/v) agar) plate containing 100 μg/mL ampicillin and 34 μg/mL chloramphenicol, then cultured overnight in LB medium (10 g/L tryptone, 5 g/L yeast extract, 10 g/L NaCl, pH7.4) with the appropriate antibiotics. Terrific broth (TB) medium (24 g/L yeast extract, 20 g/L tryptone, glycerol 4 mL/L, 100 mL/L phosphate buffer containing 0.17 M $KH_2PO_4$ and 0.72 M $K_2HPO_4$) was used to prepare a 1:100 dilution of the starter culture, and $OD_{600}$ (600 nm) was measured. At $OD_{600}$ = 1, protein induction was initiated by adding 1 mM IPTG for overnight expression at 16°C for 18 h with shaking (120 rpm). The following day, pellets were homogenized and lysed in glycerol lysis buffer (20 mM Tris-HCl pH 6.8, 300 mM NaCl, 10% (v/v) glycerol, 5 mM 2-mercaptoethanol, supplemented with Protease Inhibitor Cocktail). Lysis was performed on ice for 30 min, followed by sonication (7 × 20 sec pulses with 20 sec breaks on ice) using a Bandelin sonoplus sonicator (Sonotrode MS1.5, 70% output, 20W). Lysates were centrifuged at 4°C for 1 h at 14.000 × g, and the supernatant was transferred in a new tube. 1 L of TB growth medium culture was lysed in 20 mL of supernatant (collected in 20 × 1 mL tubes). GST beads were used according to manufacturer's instructions (Pierce™ Glutathione magnetic

agarose bead; Thermo Fisher Scientific, Waltham, MA, USA). For each 1 mL of supernatant, 100 µL of GST magnetic beads were used. 100 µL of GST magnetic beads were washed twice with 500 µL of wash buffer (125 mM Tris-HCl pH 7.4, 150 mM NaCl, 1 mM DTT, 1 mM EDTA) in a 2 mL tube. The lysis supernatant was diluted 1:1 with wash buffer, and incubated with the beads on an end-over-end rotator for 1 h at 25°C. After incubation, the supernatant was removed, and the beads were washed twice with wash buffer lacking DTT and EDTA. Protein elution was then performed by incubating the beads with glutathione elution buffer (10 mM reduced glutathione, 125 mM Tris-HCl pH 7.4, 250 mM NaCl, 0.3% Triton X-100) for 30 min at 25°C on an end-over-end rotator. This process was repeated with glutathione elution buffer containing 0.1% Triton X-100 under the same conditions. Elution fractions were pooled and concentrated by centrifugation (4°C, 25 min, 4500 rpm) using a 30 kDa cut-off concentrator (Merck Millipore, #UFC803008, Darmstadt, Germany). Purified proteins were analyzed by colloidal Coomassie stain, WB and MS.

**Streptavidin pull-downs with GST-DCAF10 and biotinylated peptides**

Streptavidin magnetic beads (Dynabeads™ MyOne™ Streptavidin T1; Thermo Fisher Scientific, Waltham, MA, USA) were washed twice with 500 µL of wash buffer (125 mM Tris-HCl pH 7.4, 150 mM NaCl, 0.1% Triton X-100). An excess of peptides was added (to ensure full occupancy of the beads) and incubated for 30 min at RT. After peptide binding, beads were again washed three times with 500 µL of wash buffer. The pull-down was then performed in a 100 µL reaction volume with final concentrations of 0.5 µM GST-DCAF10 and 5 µM peptide. GST-DCAF10 or GST alone (as a control) was added to the peptide-streptavidin beads and incubated on a rotator for 1 h at RT. After incubation, the beads were washed three times with 500 µL of wash buffer and resuspended in 4x

Laemmli buffer. 1/5 of the input (GST-DCAF10 and GST) and 9/10 of the sample were loaded on a 10% SDS gel and blotted with anti-GST and anti-DCAF10 antibodies, while 1/10 was loaded on a 15% SDS gel and blotted with anti-streptavidin antibody to assess equal peptide loading.

For direct comparison of binding to acetylated peptides (Ac-Lyn, Ac-Fyn and Ac-Src), Streptavidin Sepharose High Performance, GE Healthcare, Chicago, IL, USA) were used. After washing with a high salt wash buffer without detergents (125 mM Tris-HCl pH 7.4, 400 mM NaCl), an excess of peptides was added and incubated for 30 min at RT. For each reaction, 10 µL of slurry beads were used. After peptide-binding, beads were again washed once with a high salt wash buffer without detergents and twice with binding buffer without detergents (125 mM Tris-HCl pH 7.4, 150 mM NaCl). The pull-downs were performed in a 100 µL reaction volume in binding buffer without detergents with a final concentration of 0.5 µM GST-DCAF10 and incubated rotating for 1 h at RT. After incubation, the beads were washed five times with 500 µL of high salt wash buffer without detergents (125 mM Tris-HCl pH 7.4, 300 mM NaCl) and resuspended in 2x Laemmli buffer. 1/5 of the input (GST-DCAF10) and 9/10 of each sample were loaded on a 4–15% Mini-PROTEAN® TGX™ Precast Protein Gel (Bio-Rad, Hercules, CA, USA) and blotted with anti-GST and anti-DCAF10 antibodies, while 1/10 was loaded on a different a 4–15% Mini-PROTEAN® TGX™ Precast Protein Gel and blotted with anti-streptavidin antibody to assess equal peptide loading.

**Cloning, expression, and purification of human NatA**

Human NatA (hNatA) full-length (FL) was cloned in a pFL vector expressing both genes, NAA15 with a 6× C-term histidine tag (NAA15-6xHIS) and NAA10[2]. The expression was carried out in insect cells (Tnao38) for 72 h infected 1:20 with

baculovirus. Washed cell pellets were lysed by sonication in a lysis buffer containing 200 mM NaCl, 50 mM HEPES pH 8.0, 20 mM Imidazole, and 2 mM TCEP. The protein purification was performed as a three-step purification, starting with an affinity nickel column, followed by an ion-exchange column with a gradient of 50–300 mM NaCl, and finished with size exclusion chromatography (SEC) using an S200 10/300 column. The main peaks were collected, and the protein was concentrated in the SEC buffer (150 mM NaCl, 50 mM HEPES pH 8.0, 1 mM EDTA). The purified hNatA was flash-frozen in liquid nitrogen and stored at -80°C.

### ***In vitro*** **Nt-acetylation assay**

The method has been described in detail previously[2]. Briefly, different peptides (**Supplementary Table 1**) and concentrations (an eight-step, 1:1 dilution series for Michaelis-Menten curves) as indicated were incubated with 20 nM hNatA and 80 µM Ac-CoA in reaction buffer (50 mM HEPES pH 8.0, 0.5 mM EDTA, 0.005% CHAPS) for 60 (Src) or 120 min (Lyn), at RT in a 384-well microplate (Corning 4514) ($n$ = 4). Afterwards, the enzymatic reaction was stopped by adding 1:1 DMSO with 10 µM (final concentration 5 µM) of the thiol reactive CPM (7-Diethylamino-3-(4'Maleimidylphenyl)4-Methylcoumarin). After 1 h at RT, the readout was performed with a Tecan Spark® Multimode Microplate Reader (excitation 390 nm, emission 479 nm). Background values at 0 µM peptide were subtracted, and control reactions omitting NatA or Ac-CoA were performed. Dilution series of CoA incubated with 5 µM CPM were used to generate a linear equation (y = 10.99*x + 81.06) to calculate acetylation velocities for NatA-mediated peptide acetylation. Experiments with different time points and concentrations were performed to ensure that the reaction remained within a linear range.

### **Cloning and expression of MBP-DCAF10, StrepII-DDB1 CUL4A, and RBX1**

The cDNA sequences encoding for the human proteins DDB1 (FL), CUL4A (FL), RBX1 (FL), and DCAF10 (FL) were first cloned in a pLIB vector separately. DDB1 with an N-term cleavable (HRV 3C protease) StrepII-tag and DCAF10 were cloned into a vector containing an Nt-6xHIS-MBP fusion with a 3C protease cleavage site. Gibson cloning was performed by using pre-defined primer pairs (listed in **Supplementary Table 4**) corresponding to cassettes including each gene and combining them to one expression vector (pbiG1a)[3]. The WT construct contained cassettes I, II, and III with the genes coding for StrepII-3C-DDB1, CUL4A, and RBX1. This vector was used as a template to perform a site-directed mutagenesis to introduce two point mutations in the RBX1 gene, resulting in the change of amino acid residues cysteine 75 and histidine 77 to alanine ($RBX1^{C75A/H77A}$). Accordingly, the pLIB-Nt-6xHIS-MBP vector containing the DCAF10 WT gene was used to introduce mutations resulting in F172G, K257A and I475G. For expression of the four different constructs, the baculovirus/insect cell system was used. Therefore, pLIB and pbig1a plasmids were recombined with bacmid DNA to generate bacmids, which were then used to transfect Sf9 (*Spodoptera frugiperda*) cells and to produce high-titer V2 virus[4,5]. The virus containing the DNA for StrepII-DDB1, CUL4A, and RBX1 was co-infected with the virus containing the bacmid coding for MBP-DCAF10 at a 1:3 ratio (3× excess of DCAF10). V2 was used at a 1:50 ratio for StrepII-DDB1, CUL4A and RBX1 (WT or C75A/H77A), and at a 1:16.6 ratio for MBPDCAF10 (WT or F172G/K257A/I475G). For the expression culture, Tnao38[6,7] insect cells were used and incubated at 27°C for 72 h in a shaker.

**Purification of MBP-DCAF10, StrepII-DDB1, CUL4A, and RBX1**

Insect cells were harvested by centrifugation and a wash with PBS, before cell pellets were flash-frozen in liquid nitrogen and stored at -80ºC. For protein purification, cell pellets were resuspended in lysis buffer (50 mM HEPES pH 7.5, 250 mM NaCl, 10%

(v/v) glycerol, 2 mM TCEP, supplemented with Protease Inhibitor Cocktail), and lysed by sonication. Lysates were cleared by centrifugation at 28,000 rpm for 45 min at 4ºC using a JA-30.50 rotor (Beckman Coulter, Brea, CA, USA). The cleared and filtered (0.8 µm) lysate was applied to 1 mL of Strep-Tactin®XT 4Flow® resin (iba), pre-equilibrated once with wash buffer (100 mM Tris-HCl pH 8.0, 150 mM NaCl, 1 mM EDTA) and lysis buffer. The lysate was incubated on a tube roller for 16 h at 4ºC. After incubation, beads were washed with 50 mL of lysis buffer. Bound proteins were eluted with 10 mL of lysis buffer supplemented with 50 mM biotin, after 15 min incubation on a tube roller at 4ºC. The eluate was concentrated using 50 kDa cut-off concentrators and loaded onto a Superdex 200 10/300 or 16/60 SEC column (GE Healthcare, Chicago, IL, USA), preequilibrated in SEC buffer (250 mM Tris pH 7.5, 250 mM NaCl, 10% (v/v) glycerol, 2 mM TCEP). Fractions containing the E3 ligase complex (DCAF10, DDB1, CUL4A, and RBX1) were concentrated, flash-frozen in liquid nitrogen, and stored at -80°C. Purified proteins were visualized during and after the purification by SDS-gels containing TCE (2,2,2-trichloroethanol)[62]. The identity of proteins was confirmed by WB and MS.

***In vitro* ubiquitination assays**

The *in vitro* ubiquitination assays were performed as previously described[8]. In brief, E1 (50 nM), UBCH5B (250 nM), ubiquitin (1.5 µg), Mg-ATP (3 mM), ligation buffer, and water were pre-mixed. The premix was divided, and either CUL4A-RBX1-DDB1DCAF10 (WT), DDB1-CUL4A-RBX1$^{C75A/H77A}$-DCAF10 (mut) or CUL4A-RBX1DDB1 (-DCAF10) (250 nM each) was added. 15 µL of each premix were incubated with substrates (endogenous Lyn or Lyn-GFP constructs) bound to beads for 1 h at 30°C with shaking. Samples were then washed four to five times with a buffer containing 50 mM HEPES pH 7.5, 10% (v/v) glycerol, 150 mM NaCl, 1% Triton X-100 and 1 mM EDTA, and loaded on a gradient gel (4–15%). Ubiquitination of substrates was assessed

by WB using an anti-ubiquitin antibody. Equal substrate loading was evaluated by anti-Lyn or anti-GFP antibodies. The WBs were incubated with an anti-DCAF10 antibody (and/or anti-DDB1 or anti-CUL4A antibodies) to ensure that the E3 ligases were washed away after the reaction. Endogenous, unmodified Lyn as the substrate was prepared as follows: Dynabeads™ Protein A (10006D, Thermo Fisher Scientific, Waltham, MA, United States) were used for binding 5 µL of anti-Lyn antibody to 40 µL of bead slurry in Ab Binding & Washing Buffer (0.1 M sodium phosphate pH 8.2, 0.01% (v/v) Tween-20) for 1 h at RT. After washing once with Ab Binding & Washing Buffer (0.1 M sodium phosphate pH 8.2, 0.01% (v/v) Tween-20), beads were incubated for ca. 16 h at 4°C with 250 µg of HeLa lysate, either treated for 48 h with siRNA against NMT1/2 or control. After two washes using 500 µL low salt buffer (50 mM HEPES pH 7.5, 140 mM NaCl, 1% Triton X-100), two washes using 500 µL high salt buffer (50 mM HEPES pH 7.5, 500 mM NaCl, 1% Triton X-100), and one wash using TBS (50 mM Tris pH 7.4, 150 mM NaCl), immunoprecipitated Lyn was used as the substrate for *in vitro* ubiquitination assays.

Different versions of C-terminally GFP-fused Lyn (WT, G2A or G2P) as substrates were prepared as follows: GFP-Trap magnetic agarose beads (ChromoTek, Proteintech, Munich, Germany) were incubated with lysates from DLD-1 Lyn KO cells stably expressing inducible Lyn$^{WT}$-GFP, Lyn$^{G2A}$-GFP or Lyn$^{G2P}$-GFP. Expression was induced by adding 10–100 ng/mL doxycycline (dox) for 24 h. To obtain similar amounts of Lyn, beads were incubated with different amounts of lysates (1000, 100 or 200 µg of Lyn-GFP lysates, respectively) for ca. 16 h at 4°C. The following day, after two washes using 500 µL of low salt buffer, two washes using 500 µL of high salt buffer, and one wash using TBS, immunoprecipitated Lyn was used as the substrate for *in vitro* ubiquitination assays.

**SiRNA knockdown and overexpression experiments in HeLa, DLD-1, and RPE-1**

**cell lines**

HeLa, DLD-1 and all thereof derived stable cell lines, and RPE-1 cells were grown in Dulbecco's Modified Eagle Medium (DMEM) supplemented with 10% fetal bovine serum (FBS). Cells were counted and diluted to 100,000 cells/mL. For siRNA knockdown, the transfection reagent Lipofectamine RNAiMAX (Invitrogen, Thermo Fisher Scientific, Waltham, MA, USA) was used at a final dilution of 1:333 (3 µL/mL of medium). Pools of siRNAs, including siNMT1, siNMT2, siDCAF10, siZYG11B, and siZER1, or combinations of different siRNAs, were reverse transfected at a concentration of 25 nM for each siRNA and incubated as indicated (48–72 h). As control, cells were treated only with Lipofectamine RNAiMAX. Both siRNAs and RNAiMAX were diluted in Opti-MEM (Thermo Fisher Scientific, Waltham, MA, USA). For overexpression of DCAF10, the transfection reagent Lipofectamine 2000 (Thermo Fisher Scientific, Waltham, MA, USA) was used at a final dilution of 1:200. For one 6-well of a 6-well plate, 4 µg of DNA were combined with Lipofectamine 2000 and diluted in Opti-MEM. The medium was changed 4–6 h post-transfection. The transfection was performed one day after plating, and the cells were incubated for an additional 24 h. For proteasomal or translational inhibition, the cells were treated with 10 µM MG132 or 100 µg/mL cycloheximide for 4 and 8 h before lysis, respectively.

**High-pH fractionation for DCAF10 and ZYG11B**

10 µg of cellular lysates were reduced, alkylated, and digested with LysC/Trypsin (protein:enzyme ratio of 1:100) according to MS standard in-solution digest[9]. Peptides were then separated with a high-pH fractionation kit (eight fractions for DCAF10 and four fractions for ZYG11B; Thermo Fisher Scientific, Waltham, MA, USA). Each fraction was measured by MS (see **Mass spectrometry measurements and raw file processing** for settings).

**Site-directed mutagenesis**

To generate Lyn variants with mutated N-terminal amino acid (G2A and G2P), site-directed mutagenesis was performed on the pcDNA5/FRT/TO-Lyn$^{WT}$-EGFP-IRES construct using primers (listed in **Supplementary Table 4**) that carry single (for G2A) or double (for G2P) mismatches to wild-type Lyn in PCR reactions. The resulting PCR products were purified using the Wizard® SV Gel and PCR Clean-Up System (Promega; Madison, WI, USA) according to the manufacturer's instructions, followed by DpnI digestion, phosphorylation, and ligation. Subsequently, half of the ligation reaction was transformed into DH5α competent cells. After isolation, the plasmid DNA from single colonies was sent for Sanger sequencing (Eurofins Genomics, Munich, Germany) to confirm successful mutagenesis.

**Generation of DLD-1 Lyn KO Flp-In T-REx cell lines**

DLD-1 Lyn KO Flp-In T-REx cells were generated using two guide RNAs (gRNAs), each containing a specific crRNA (listed in **Supplementary Table 4**) and a tracrRNA. To form each Cas9-gRNA ribonucleoprotein (RNP), a gRNA was mixed with the homemade wild-type Cas9 (prepared following the procedure described in[10]). The electroporation method using SF Cell Line 4D-Nucleofector X Kit S (Lonza Bioscience, Basel, Switzerland) was applied to transfect the Cas9-gRNA RNP into cells. Briefly, 250,000 cells were resuspended in 20 μL of SF Full Electroporation Buffer containing 16.4 μL of SF Nucleofector Solution and 3.6 μL of Supplement. Next, 1.25 μL of Cas9 Electroporation Enhancer (IDT) and 3.75 μL of Nuclease Free Duplex Buffer were added to the cell suspension, followed by addition of 5 μL of the RNP. The mixture was then transferred to a free well of a 16-well Nucleocuvette strip. The strip was placed into the 4DNucleofector System (Lonza Bioscience, Basel, Switzerland), and cells were

electroporated using the CM137 program. Following electroporation, the cells were kept in the strip at RT for 10 min before being transferred to a 6-well plate containing prewarmed DMEM supplemented with 10% FBS and 100 U/mL penicillin/streptomycin. After a day, all cells from the 6-well plate were transferred to a 10-cm dish. After two days, single cells were diluted into each well of a 96-well plate using a single cell dispenser (Dispencell; SEED Biosciences, Épalinges, Vaud, Switzerland) following the manufacturer's instructions. The 96-well plate was incubated for two weeks, after which cells were expanded in a 24-well plate and a 6-well plate until clones reached 80% confluency. Once clones reached 80% confluency in 6-well plates, they were harvested. One third of the cells from each clone were frozen, and the remaining cells were analyzed by genomic sequencing (primers in **Supplementary Table 4**), WB and MS. Positive clones were then expanded for cell line generation.

### Integration of $Lyn^{WT}$-GFP and N-terminal mutants, $Lyn^{G2A}$-GFP and $Lyn^{G2P}$-GFP, in DLD-1 Lyn KO Flp-In T-REx cell lines

Stable cell lines expressing C-terminally GFP-fused Lyn, either WT, G2A or G2P mutants, were generated in DLD-1 Lyn KO Flp-In T-REx cells using FRT/Flp-mediated recombination technique[52]. One day prior to transfection, cells were seeded in 10-cm dishes to reach a confluency of 50% on the day of transfection. For each transfection reaction, 30 µL of X-tremeGENE transfection reagent (Roche, Basel, Switzerland) were mixed with 500 µL of pre-warmed Opti-MEM and incubated for 5 min at RT. In the meantime, 1 µg of the pCDNA5/FRT/TO-EGFP-IRES vector containing the respective Lyn cDNA (WT, G2A or G2P) was mixed with 9 µg of pOG44 and added to the X-tremeGENE mix. After 20 min incubation at RT, the transfection mix was added dropwise to the cells, and cells were cultured for 48 h. Next, cells were subjected to selection in DMEM supplemented with 10% FBS, 300 µg/mL hygromycin B, and 8 µg/mL blasticidin

for two weeks. Resulting foci were pooled, expanded, and validated for expression of the transgenes by WB and fluorescence microscopy. The inducible gene expression was tested by addition of 10–100 ng/mL dox for 8–24 h.

**Immunoprecipitation of Lyn$^{WT}$-GFP**

Lyn$^{WT}$-GFP was immunoprecipitated from lysates of DLD-1 Lyn KO Flp-In T-REx cells expressing inducible Lyn$^{WT}$-GFP, following 72 h NMT1/2 knockdown and 24 h dox induction using GFP-Trap magnetic agarose beads. Briefly, 10 µL of bead slurry per reaction was equilibrated three times with ice-cold lysis buffer (75 mM HEPES pH 7.5, 150 mM KCl, 1.5 mM EGTA pH 8.0, 1.5 mM MgCl2, 10% (v/v) glycerol, 0.075% NP40, supplemented with 1mM DTT, PhosSTOP, and Protease Inhibitor Cocktail). Then, 500 µL of each lysate sample (control, dox induction, and NMT1/2 knockdown with or without dox induction) containing approximately 300 µg total protein, were added to tubes containing the equilibrated beads. Samples were rotated end-over-end for 1.5–2 h at 4°C. After incubation, the beads were washed twice with 500 µL of cold lysis buffer and twice with 500 µL of cold lysis buffer lacking NP-40, glycerol, DTT, phosSTOP, and Protease Inhibitor Cocktail. Finally, the immunoprecipitates were resuspended in 50 µL of 8 M urea, incubated at 25°C for 30 min, and stored at -20°C prior to on-bead digestion and stage tip clean-up for MS analysis. Each experimental condition was performed in triplicates.

**Fluorescence microscopy**

Cells were seeded on coverslips to be 90% confluent on the day of fixation. Lyn-GFP expression was induced by 24 h dox treatment (100 ng/mL for Lyn$^{WT}$, and 10 ng/mL for Lyn$^{G2A}$ and Lyn$^{G2P}$), or media was just replaced for the controls (without dox). Cells were washed once with PBS before fixation with 4% PFA in PBS for 10 min at RT. After three

washes with PBS, cells were permeabilized with PBS-T (PBS with 0.1% (v/v) Tween20) for 10 min at RT. Afterwards, cover slips were incubated in 0.5 µg/mL DAPI diluted in PBS-T for 5 min at RT. After a final wash in dd$H_2O$, the coverslips were dried on the air and mounted with Mowiol as a mounting medium on glass slides. Image acquisition of cells was performed at RT using a Deltavision Elite System (GE Healthcare, Chicago, IL, USA) equipped with an IX-71 inverted microscope (Olympus, Tokyo, Japan), a 60x/1.42 Plan Apo N objective and a pco.edge sCMOS camera (PCO-TECH Inc., Kelheim, Germany). Images were acquired as z-stacks containing 16 sections with a distance of 200 nm using the software softWoRx (GE Healthcare, Chicago, IL, USA). Raw data were then deconvolved and converted into average intensity projections, exported, and saved as 16-bit TIFF files. Figures were edited and arranged using ImageJ. Acquisition settings: Green channel: 0.1 sec, 50% transmission; DAPI channel: 0.01 sec, 32% transmission.

**Mass spectrometry measurements and raw file processing**

Samples (peptide pull-downs, IPs and high-pH fractionations) were separated on an EASY-nLC 1200 HPLC system (Thermo Fisher Scientific, Waltham, MA, USA) using in-house-packed 50-cm reversed-phase columns (75 cm diameter, 1.9 mm C18 ReproSil particles, Dr. Maisch GmbH) (60°C at 300 nL/min flow rate with an 83 min gradient from 5–60% acetonitrile with 0.1% formic acid). Peptides were directly sprayed via a nano electrospray source in an Orbitrap Exploris™ 480 (Thermo Fisher Scientific, Waltham, MA, USA). Data were acquired in a data-independent mode (DIA), acquiring one survey scan (MS scan) with 120 k resolution and subsequently 85 windows with an isolation width of 7.7 Th with 1 Dalton overlap from 350 to 1000 m/z at a resolution of 15 k (3.2 sec cycle time) (data points per peak = 6). Cycle times were calculated based on the median elution time of HeLa QC runs to obtain 6 data points. The target value was set to

300% for the MS scan and 1000% for the MS/MS scans. The maximum injection time was 45 msec for the MS scans. HCD Collision energy was set to 30%.

**Data Analysis and statistics**

Resulting raw files from MS measurements were processed with DIA-NN (version 1.8.2 beta 22) using a UniProt human database (reference January 2023) or UniProt mouse database (reference January 2023)[53]. Oxidation (M), M excision, Nt-acetylation, and carbamidomethylation (C) were given as modifications. One miscleavage and two modifications per peptide were allowed. A false discovery rate cutoff of 1% was applied. MS measurements were performed in triplicates for peptide pull-downs, whole proteome analysis and IPs. Label-free quantifications were calculated by the integrated DIA-NN algorithm[11].

Peptide pull-downs from Ac and free N-termini in **Fig. 1** and **Supplementary Fig. 1** were analyzed altogether; Ac and Myr, swap and Ala-Ala-Ala mutants, and Nt-Lyn mutants were analyzed together, respectively. $Lyn^{WT}$-GFP IPs were analyzed together and high pH fractionations of DCAF10 and Zyg11B. Comparisons for IPs and peptide pull-downs were performed pairwise, filtering for 3 out of 3 replicates in one condition and subsequent data imputation (downshift: 1.8, width: 0.3) in Perseus[12].

Intensities of high-pH fractions from the report.pg_matrix.tsv were then summed and $\log_2$-transformed ($\log_2$) for overall protein quantities. DCAF10 and ZYG11B were detected in control cells but not in KD cells. To not overestimate the KD, we considered that the protein amounts of ZYG11B and ZER in the siRNA treated cells must be below the detection limit of these MS runs. Therefore, we used for our knock-down estimation the $\log_2$ intensity of DCAF10 or ZYG11B, respectively, in the control cells and subtracted it from the lowest quantified protein. For DCAF10: 22.37 (mean $\log_2$ intensity of DCAF10) - 14.35 ($\log_2$ intensity of the lowest quantified protein) means at least a $\log_2$

reduction of 8. For ZYG11B: 19.7 (mean $log_2$ intensity of Zyg11B) - 15.7 ($log_2$ intensity of lowest quantified protein) means at least a $log_2$ reduction of 4.

## Supplementary Material Tables

Peptides have been purchased from GenScript (Piscataway, NJ, USA).

**Supplementary Table 1. Peptides**

| Gene name | Peptide sequence | Protein name / UniProt ID | N-terminal modification | C-terminal modification |
|---|---|---|---|---|
| ARF1 | GNIFANLFKGKK | ADP-ribosylation factor 1 / P84077 | / | K(biotin) |
| ARF1 | GNIFANLFKGKK | ADP-ribosylation factor 1 / P84077 | acetyl-group | K(biotin) |
| COX17 | PGLVDSNPAPKK | Cytochrome c oxidase copper chaperone / Q14061 | / | K(biotin) |
| EF1B | GFGDLKSPAGKK | Elongation factor 1-beta / P24534 | / | K(biotin) |
| EF1B | GFGDLKSPAGKK | Elongation factor 1-beta / P24534 | acetyl-group | K(biotin) |
| FYN | GCVQCKDKEAKK | Tyrosine-protein kinase Fyn / P06241 | / | K(biotin) |
| FYN | GCVQCKDKEAKK | Tyrosine-protein kinase Fyn / P06241 | acetyl-group | K(biotin) |
| FYN | GCVQCKDKEAKK | Tyrosine-protein kinase Fyn / P06241 | myristoyl-group | K(biotin) |
| GNAI3 | GCTLSAEDKAKK | Guanine nucleotidebinding protein G(i) subunit alpha-3 / P08754 | / | K(biotin) |
| GNAI3 | GCTLSAEDKAKK | Guanine nucleotidebinding protein G(i) subunit alpha-3 / P08754 | acetyl-group | K(biotin) |
| LYN | GCIKSKGKDSKK | Tyrosine-protein kinase Lyn / P07948 | / | K(biotin) |
| LYN | GCIKSKGKDSKK | Tyrosine-protein kinase Lyn / P07948 | acetyl-group | K(biotin) |
| LYN | GCIKSKGKDSKK | Tyrosine-protein kinase Lyn / P07948 | myristoyl-group | K(biotin) |
| LYN G2A | ACIKSKGKDSKK | Tyrosine-protein kinase Lyn / P07948 | acetyl-group | K(biotin) |
| LYN G2P | PCIKSKGKDSKK | Tyrosine-protein kinase Lyn / P07948 | / | K(biotin) |
| LYN AAA | GCIAAAGKDSKK | Tyrosine-protein kinase Lyn / P07948 | acetyl-group | K(biotin) |

| | | | | |
|---|---|---|---|---|
| LYN TDD | GCITDDGKDSKK | Tyrosine-protein kinase Lyn / P07948 | acetyl-group | K(biotin) |
| *NatA target peptide* | SASEAGVRWGRP VGRRRR | / | / | / |
| NDUFAF4 | GALVIRGIRNKK | NADH dehydrogenase [ubiquinone] 1 alpha subcomplex assembly factor 4 / Q9P032 | / | K(biotin) |
| NDUFAF4 | GALVIRGIRNKK | NADH dehydrogenase [ubiquinone] 1 alpha subcomplex assembly factor 4 / Q9P032 | acetyl-group | K(biotin) |
| SRC | GSNKSKPKDAKK | Proto-oncogene tyrosineprotein kinase Src / P12931 | / | K(biotin) |
| SRC | GSNKSKPKDAKK | Proto-oncogene tyrosineprotein kinase Src / P12931 | acetyl-group | K(biotin) |
| SRC | GSNKSKPKDAKK | Proto-oncogene tyrosineprotein kinase Src / P12931 | myristoyl-group | K(biotin) |
| THOC7 | GAVTDDEVIRKK | THO complex subunit 7 / Q6I9Y2 | / | K(biotin) |
| THOC7 | GAVTDDEVIRKK | THO complex subunit 7 / Q6I9Y2 | acetyl-group | K(biotin) |
| THOC7 KSK | GAVKSKEVIRKK | THO complex subunit 7 / Q6I9Y2 | acetyl-group | K(biotin) |
| TMEM97 | GAPATRRCVEKK | Sigma intracellular receptor 2 / Q5BJF2 | / | K(biotin) |
| TMEM97 | GAPATRRCVEKK | Sigma intracellular receptor 2 / Q5BJF2 | acetyl-group | K(biotin) |
| YES | GCIKSKENKSKK | Tyrosine-protein kinase Yes / P07947 | / | K(biotin) |
| YES | GCIKSKENKSKK | Tyrosine-protein kinase Yes / P07947 | acetyl-group | K(biotin) |

**Supplementary Table 2. Cell lines**

| **Cell line** | **Plasmid** | **Gene** | **Tag** | **Reference** |
|---|---|---|---|---|
| DLD-1 Flp-In™ T-REx™ Lyn KO | / | / | / | this study |
| DLD-1 KO Lyn Flp-In™ T-REx™ Lyn$^{G2A}$-GFP | pCDNA5/FRT /TO-EGFP- | LYN- G2A | GFP (C-term) | this study |

| | | | | |
|---|---|---|---|---|
| | IRES | | | |
| DLD-1 Flp-In™ T-REx™ | / | / | / | A gift from AG Musacchio, Dr. Stefano Maffini (MPI Dortmund) |
| DLD-1 Flp-In™ T-REx™ Lyn KO | / | / | / | this study |
| DLD-1 KO Lyn Flp-In™ T-REx™ Lyn$^{G2A}$-GFP | pCDNA5/FRT /TO-EGFP-IRES | LYN- G2A | GFP (C-term) | this study |
| DLD-1 KO Lyn Flp-In™ T-REx™ Lyn$^{G2P}$-GFP | pCDNA5/FRT /TO-EGFP-IRES | LYN- G2P | GFP (C-term) | this study |
| DLD-1 KO Lyn Flp-In™ T-REx™ Lyn$^{WT}$-GFP | pCDNA5/FRT /TO-EGFP-IRES | LYN- WT | GFP (C-term) | this study |
| Hela Flp-In™ T-REx™ | / | | | A gift from S. Taylor (University of Manchester) |
| RPE-1 Flp-In™ T-REx™ | / | | | A gift from AG Musacchio, Dr. Stefano Maffini (MPI Dortmund) |

**Supplementary Table 3. siRNAs**

| siRNA | Company | Identifier |
|---|---|---|
| ON-TARGETplus siRNA Human DCAF10 | Dharmacon | L-014344-01-0005 |
| ON-TARGETplus siRNA Human NMT1 | Dharmacon | L-004316-01-0005 |
| ON-TARGETplus siRNA Human NMT2 | Dharmacon | L-004317-01-0005 |
| ON-TARGETplus siRNA Human ZER1 | Dharmacon | L-019424-02-0005 |
| ON-TARGETplus siRNA Human ZYG11B | Dharmacon | L-021798-02-0005 |

**Supplementary Table 4. Oligonucleotides and primers (F: Forward, R: Reverse)**

| Sequence 5´to 3´ | Company | Purpose |
|---|---|---|
| GTTCTGTTCCAGGGGCCCCTGGGATCCATGTTTCCCTTTGGGCCCCATAG | Metabion | F primer DCAF10 to clone in pGEX6P-2rbs for Gibson cloning |
| CAGTCAGTCACGATGCGGCCGCTCGAGCTACTAAAACTTTGGCTGTACAAAGAAACCCG | Metabion | R primer DCAF10 to clone in pGEX6P-2rbs for Gibson cloning |
| TTCTTCCAGTTGCCCCCTCT | Metabion | crRNA for Lyn KO (sequence 1) |
| GGAGGCCTCATACCCATTACA | Metabion | crRNA for LYN KO (sequence 2) |

| | | |
|---|---|---|
| GCCACCATGGTGAGCAAG | Metabion | pcDNA5-F to linearize pcDNA5/FRT/TO-EGFP-IRES |
| GGTACCAAGCTTAAGTTTAAACGC | Metabion | pcDNA5-R to linearize pcDNA5/FRT/TO-EGFP-IRES |
| TTAAACTTAAGCTTGGTACCATGGGATG TATAAAATCAAAAGGG | Metabion | F primer to amplify LYN cDNA from pCR4-TOPO-LYN for Gibson cloning |
| CCCTTGCTCACCATGGTGGCAGGCTGCT GCTGGTATTG | Metabion | R primer to amplify LYN cDNA from pCR4-TOPO-LYN for Gibson cloning |
| CTTAAGTTTAAACGCTAGAGTCCG | Metabion | LYN-R Site-directed mutagenesis of LYN in the pcDNA5/FRT/TOLYN$^{WT}$-EGFP-IRES construct |
| CTTGGTACCATGGCATGTATAAAATC | Metabion | LYN G2A-F Site-directed mutagenesis of LYN in the pcDNA5/FRT/TO-LYN$^{WT}$-EGFP-IRES construct |
| CTTGGTACCATGCCATGTATAAAATC | Metabion | LYN G2P-F Site-directed mutagenesis of LYN in the pcDNA5/FRT/TOLYN$^{WT}$-EGFP-IRES construct |
| GCTACCCGGGATGAACATCCGCAATGC C | Sigma | NAA10 F - Amplifying full-length wildtype human NAA10 for pFH vector |
| TGCAGCTAGCTTAGCTGGCGCTATCGC | Sigma | NAA10 R - Amplifying full-length wildtype human NAA10 for pFH vector |
| GATCGGATCCATGCCAGCCGTGTCCCTG | Sigma | NAA15 F - Amplifying full-length wildtype human NAA15-6xHIS for pFH vector |
| CGTAGTCGACCTAGTGATGGTGATGGTG ATGGATCTCGTTGGCCAGCTC | Sigma | NAA15-6xHIS R - Amplifying fulllength wildtype human NAA156xHIS for pFH vector |
| TTGTTTCAAGGTCCTGGATCCTCGTACA ACTACGTGGTAACGGC | Sigma | DDB1 F StrepII - Gibson cloning in pLIB |
| TCCTCTAGTACTTCTCGACAAGCTTTTA CTAATGGATCCGAGTTAGCTCCTCC | Sigma | DDB1 R - Gibson cloning in pLIB |
| CCACCATCGGGCGCGGATCCATGCATCA CCATCACCATCACTTTCCCTTTGGGCCC CATAGC | Sigma | DCAF10 6xHIS F - Gibson cloning in pLIB |
| CTGTTCCAGGGGCCCGGATCCTTTCCCT TTGGGCCCCATAGC | Sigma | DCAF10 F MBP - Gibson cloning in pLIB |
| TCCTCTAGTACTTCTCGACAAGCTTTTAT CAAAACTTTGGCTGATACAAAGAAACC CG | Sigma | DCAF10 R - Gibson cloning in pLIB |
| CCACCATCGGGCGCGGATCCATGGCG GACGAGGCCC | Sigma | CUL4A F - Gibson cloning in pLIB |

| | | |
|---|---|---|
| TCCTCTAGTACTTCTCGACAAGCTTTTAT CAGGCCACGTAGTGGTACTG | Sigma | CUL4A R - Gibson cloning in pLIB |
| CCACCATCGGGCGCGGATCCATGGCGG CGGCGATG | Sigma | RBX1 F - Gibson cloning in pbig1a |
| TCCTCTAGTACTTCTCGACAAGCTTTTA CTAATGCCCATACTTCTGGAACTCCC | Sigma | RBX1 R - Gibson cloning in pbig1a |
| AACGCTCTATGGTCTAAAGATTTAAATC GACCTACTCCGGAATATTAATAGATC | Sigma | Cas I F - Gibson cloning in pbig1a |
| AAACGTGCAATAGTATCCAGTTTATTTA AATGGTTATGATAGTTATTGCTCAGCG | Sigma | Cas I R - Gibson cloning in pbig1a |
| AAACTGGATACTATTGCACGTTTAAATC GACCTACTCCGGAATATTAATAGATC | Sigma | Cas II F - Gibson cloning in pbig1a |
| AAACATCAGGCATCATTAGGTTTATTTA AATGGTTATGATAGTTATTGCTCAGCG | Sigma | Cas II R - Gibson cloning in pbig1a |
| AAACCTAATGATGCCTGATGTTTAAATC GACCTACTCCGGAATATTAATAGATC | Sigma | Cas III F - Gibson cloning in pbig1a |
| AACCCCGATTGAGATATAGATTTATTTA AATGGTTATGATAGTTATTGCTCAGCG | Sigma | Ω R - Gibson cloning in pbig1a |
| GTACGGTTGCATGGGGAGTCGCCAACG CTGCTTTTCATTTCCACTGCATC | Sigma | RBX1 mut C75A/H77A - Gibson cloning in pbig1a |
| ATTGGAGACATTTTGATGGCTTG | Metabion | F - Diagnostic primers for LYN KO exon 2 |
| TTTCTTCACACAAAAGAATGTGACC | Metabion | R - Diagnostic primers for LYN KO exon 2 |
| TTCTTCCAGTTGCCCCCTCT | Metabion | F - Diagnostic primers for LYN KO exon 4 |
| GGAGGCCTCATACCCATTACA | Metabion | R - Diagnostic primers for LYN KO exon 4 |
| CACCCACGGCGCCGTCGGCAACCTCGAG TACTC | Eurofins | DCAF10 mut F172G |
| CACACTAGCTGGGTGGCGAACATCGAATA TGATAC | Eurofins | DCAF10 mut K257A |
| GTAGGCAGGGGTTACGGCAAAGAACTTT GCTTC | Eurofins | DCAF10 mut I475G |

**Supplementary Table 5.** **Plasmids**

| **Plasmid backbone** | **Gene** | **Reference** |
|---|---|---|
| pbiG1a | / | A gift from AG Musacchio, (MPI Dortmund) |
| pbiG1a | DDB1, CUL4A, RBX1 human | this study |
| pbiG1a | DDB1, CUL4A, RBX1$^{C75A/H77A}$ human | this study |
| pcDNA3.1+/c-(k)-DYK | DCAF10 human | Genscript ORF #OHu10624D |
| pcDNA5/FRT/TO--EGFP-IRES | LYN$^{G2A}$ human | this study |
| pCDNA5/FRT/TO-EGFP-IRES | / | A gift from AG Musacchio, Dr. Stefano Maffini (MPI Dortmund) |
| pcDNA5/FRT/TO-EGFP-IRES | LYN$^{WT}$ human | this study |
| pcDNA5/FRT/TO-EGFP-IRES | LYN$^{G2P}$ human | this study |

| | | |
|---|---|---|
| pCR4-TOPO-LYN | LYN human | Horizon Discovery/Dharmacon (#MHS6278-202856900) |
| pFL | / | A gift from AG Musacchio (MPI Dortmund) |
| pFL | NAA15-6xHIS, NAA10 human | [2] |
| pGEX-6P-2rbs | DCAF10 human | this study |
| pGEX-6P-2rps | / | GenBank accession code KM817768 |
| pLIB | / | A gift from AG Musacchio (MPI Dortmund) |
| pLIB | CUL4A human | this study |
| pLIB | RBX1 human | this study |
| pLIB-6xHis-MBP | DCAF10 human | this study |
| pLIB-6xHis-MBP | $DCAF10^{F172G/K257A/I475G}$ human | this study |
| pLIB-HIS-MBP | / | A gift from AG Musacchio, Dr. John Weir (MPI Dortmund) |
| pLIB-StrepII | DDB1 human | this study |
| pLIB-StrepII-3C | / | A gift from AG Musacchio (MPI Dortmund) |
| pOG44 | / | A gift from AG Musacchio, Dr. Stefano Maffini (MPI Dortmund) |

**Supplementary Table 6. Antibodies**

| **Antibody** | **Company** | **Identifier** | **Dilution** |
|---|---|---|---|
| anti-CUL4A Rabbit pAb | Cell Signaling | #2699 | 1:1000 |
| anti-DCAF10/ WDR32 Rabbit pAb | Thermo Fisher Scientific | #PA5-24133 | 1:1000 |
| anti-DDB1 (D4C8) Rabbit mAb | Cell Signalling | #6998 | 1:1000 |
| anti-FLAG (DYKDDDK) Mouse mAb | Cell Signaling | #8146S | 1:5000 |
| anti-Fyn Rabbit pAb | Cell Signaling | #4023S | 1:1000 |
| anti-GFP Rabbit pAb | In-house | AG Musacchio | 1:1000 |
| anti-GFP Mouse mAb | Roche | #11814460001 | 1:10000 |
| anti-GST Mouse mAb | Merck Millipore | #71097 | 1:1000 |
| anti-Lyn (C13F9) Rabbit mAb | Cell Signaling | #2796S | 1:1000 |
| anti-mouse IgG HRP linked antibody | Cell Signaling | #CST 7076S | 1:10000 |
| anti-NMT1 Rabbit pAb | abcam | #AB186123 | 1:1000 |
| anti-NMT2 Rabbit pAb | abcam | #AB230028 | 1:1000 |
| anti-rabbit IgG HRP-linked Antibody | Cell Signaling | #7074S | 1:10000 |
| anti-Src (36D10) Rabbit mAb | Cell Signaling | #2109S | 1:1000 |
| anti-Streptavidin Rabbit pAb | Rockland immunochemicals | #100-4195 | 1:10000 |

| | | | |
|---|---|---|---|
| anti-THOC7 Rabbit pAb | abcam | #ab155218 | 1:500 |
| anti-Ubiquitin-HRP (P4D1) Mouse mAb | Cell Signaling | #14049 | 1:1000 |
| anti-Vinculin Mouse mAb | Sigma | #V9131 | 1:10000 |
| anti-ZER1 Rabbit pAb | Proteintech | #16647-1-AP | 1:1000 |
| anti-ZYG11B Rabbit pAb | antibodies-online.com | #ABIN4916715 | 1:500 |
| VeriBlot Detection Reagent (for IP samples) | abcam | #ab131366 | 1:500 |

**Supplementary Table 7. Antibiotics used in bacterial and cell culture**

| Reagent | Working concentration | Company |
|---|---|---|
| Ampicillin | 100 µg/mL | Sigma |
| Blasticidin | 8 µg/mL | Sigma |
| Chloramphenicol | 34 µg/mL | Sigma |
| Doxycycline | 10–100 ng/mL | Sigma |
| Gentamicin | 10 µg/mL | Sigma |
| Hygromycin B | 400 µg/mL | Sigma |
| Kanamycin | 50 µg/mL | Sigma |
| Penicillin-streptomycin | 100 U/mL | Gibco/Thermo Fisher Scientific |
| Tetracyclin | 7 µg/mL | Sigma |

**Supplementary Table 8. Reagents and Kits**

| Reagent | Company | Reference number |
|---|---|---|
| Acetyl-Coenzym A | Sigma Aldrich | A2056 |
| Biotin | iba | 2-1016-002 |
| BL21(DE3) | Thermo Fisher Scientific | EC0114 |
| CoA | Sigma Aldrich | C4282 |
| CPM (7-Diethylamino-3-(4'-Maleimidylphenyl)-4-Methylcoumarin) | Invitrogen | D346 |
| Cycloheximide | Sigma Aldrich | C4859-1mL |
| Dulbecco's Modified Eagle Medium (DMEM), high glucose, GlutaMAX Supplement, pyruvate | Gibco/Thermo Fisher Scientific | 31966047 |
| Dynabeads ™ MyOne™ Streptavidin | Invitrogen/ Thermo Fisher Scientific | 65601 |
| Dynabeads™ Protein A Immunoprecipitation Kit | Invitrogen/ThermoFisher Scientific | 10006D |
| Fetal bovine serum (FBS) | Pan Biotech | P13-3001 |
| GeneJET Plasmid Miniprep Kit | Thermo Fisher Scientific | K0502 |
| GFP-Trap magnetic agarose | ChromoTek | gtma |
| Gluthatione | Thermo Fisher Scientific | 78259 |
| GST magnetic agarose beads | Pierce/ Thermo Fisher Scientific | #78602 |

| | | |
|---|---|---|
| IPTG | Thermo Fisher Scientific | 15529019 |
| Lipofectamine RNAiMAX | Invitrogen/ Thermo Fisher Scientific | 13778-150 |
| Lipofectamine® 2000 | Invitrogen/ Thermo Fisher Scientific | 11668-027 |
| LysC | Wako Chemicals | 129-02541 |
| MG132 | Sigma-Aldrich | 474787 |
| Monarch DNA Gel Extraction Kit | New England BioLabs | T1120S |
| Monarch PCR & DNA Cleanup Kit | New England BioLabs | T1130S/L |
| Opti-MEM I reduced-Serum medium | Gibco/Thermo Fisher Scientific | 31985062 |
| PageRuler Plus Prestained Protein Ladder | Thermo Fisher Scientific | 26619 |
| Phsophostop | Roche | 04906845001 |
| Pierce ™ Glutathione Magnetic Agarose | Thermo Fisher Scientific | 78602 |
| Precision Plus Protein Standard Dual color | BIO-RAD | 1610374 |
| Protease Inhibitors | Roche | 04693132001 |
| PureYield Plasmid Miniprep System | Promega | A1223 |
| Recombinant Human UbcH5a/UBE2D1 Protein (E2) | Bio-Techne GmbH | E2-622 |
| Recombinant Human Ubiquitin Activating Enzyme (UBE1) | Bio-Techne GmbH | E-304-050 |
| Recombinant Human Ubiquitin Protein (Ub) | Bio-Techne GmbH | P0CG47.1 |
| SF Cell Line 4D-Nucleofector X Kit S | Lonza | V4XC-2032 |
| Strep-Tactin®XT 4Flow® beads | iba | 2-5010-010 |
| Trypsine | Sigma-Aldrich | T6567 |
| Wizard SV Gel and PCR Clean-Up System | Promega | A9281 |
| X-tremeGENE 360 Transfection reagent | Sigma-Aldrich | XTG360RO |

**Supplementary Table 9. List of enzymes**

| **Enzyme** | **Supplier** | **Reference Number** |
|---|---|---|
| *BamH*I HF | New England Biolabs GmbH | R3136S |
| Benzonase® Nuclease | Sigma-Aldrich | 9025-65-4 |
| *Dpn*I restriction enzyme | New England Biolabs GmbH | RO176S |
| Gibson Cloning Master Mix (containing T5 Exonuclease, Phusion polymerase, Tag ligase) | New England Biolabs GmbH/*inhouse* AG Musacchio | E2611S |
| *Hind*III HF | New England Biolabs GmbH | R3104S |
| Phusion® High-Fidelity PCR 2x Master Mix | New England Biolabs GmbH | M0530S |
| *Pme*I | New England Biolabs GmbH | R0560S |
| Q5® High-Fidelity 2x Master Mix | New England Biolabs GmbH | M0494S |
| *Swa*I | New England Biolabs GmbH | R0604S |
| *T4 DNA Ligase* | New England Biolabs GmbH | M0202S |
| *T4 Polynucleotide Kinase* | New England Biolabs GmbH | M0201S |

**Supplementary Table 10. Software and Algorithms**

| **Software** | **Version** | **Supplier** |
|---|---|---|
| BioRender | - | BioRender |
| DIA-NN | 1.8.2. beta 22 and 27 | https://github.com/vdemichev/DiaNN[11] |
| GraphPad Prism | Version 8.4.3 | GraphPad Software, LLC. |
| ImageJ | 2.0.0-rc-69/1.52p | National Institutes of Health |
| Image Lab Software | 6.0.1 | BIO-RAD |
| Maxquant | 1.6.14, 2.0.3.1 | https://www.maxquant.org/[12] |
| Perseus | 1.6.50, 2.0.11 | https://maxquant.net/perseus/[11] |
| PyMOL: The PyMOL molecular Graphics System | Version 3.0 | Schrödinger, LLC |
| Resolve3D softWoRx-Acquire | Version: 7.2.0 | Release RC4 |
| Rstudio | version 2023.06.0 | Posit Software, PBC |
| SnapGene | 7.1.2 | GSL Biotech LLC |
| AF3 | 3.0.0 | [13] |

## Supplementary References


1. Müller, F. & Bange, T. Identification of N-degrons and N-recognins using peptide pull-downs combined with quantitative mass spectrometry. *Methods Enzymol.* **686**, 67–97 (2023).

2. Mueller, F. *et al.* A fluorescent CPM-based in vitro acetylation assay: A tool for assessing N-terminal acetyltransferase activity and profiling compound activity. *Methods Enzymol.* **718**, 51–85 (2025).

3. Gibson, D. G. *et al.* Enzymatic assembly of DNA molecules up to several hundred kilobases. *Nat. Methods* **6**, 343–345 (2009).

4. Volkov, V. A., Huis in ’t Veld, P. J., Dogterom, M. & Musacchio, A. Multivalency of NDC80 in the outer kinetochore is essential to track shortening microtubules and generate forces. *eLife* **7**, e36764 (2018).

5. Weissmann, F. *et al.* biGBac enables rapid gene assembly for the expression of large multisubunit protein complexes. *Proc. Natl. Acad. Sci.* **113**, E2564–E2569 (2016).

6. Hashimoto, Y., Zhang, S. & Blissard, G. W. Ao38, a new cell line from eggs of the black witch moth, Ascalapha odorata (Lepidoptera: Noctuidae), is permissive for AcMNPV infection and produces high levels of recombinant proteins. *BMC Biotechnol.* **10**, 50 (2010).

7. Hashimoto, Y., Zhang, S., Zhang, S., Chen, Y.-R. & Blissard, G. W. Correction:BTI-Tnao38, a new cell line derived from Trichoplusia ni, is permissive for AcMNPV infection and produces high levels of recombinant proteins. *BMC Biotechnol.* **12**, 12 (2012).

8. Mueller, F. *et al.* Overlap of NatA and IAP substrates implicates N-terminal acetylation in protein stabilization. *Sci Adv* **7**, (2021).

9. Shevchenko, A., Tomas, H., Havli, J., Olsen, J. V. & Mann, M. In-gel digestion for mass spectrometric characterization of proteins and proteomes. *Nat. Protoc.* **1**, 2856–2860 (2006).

10. Anders, C. & Jinek, M. *In Vitro* Enzymology of Cas9. in *Methods in Enzymology* (eds Doudna, J. A. & Sontheimer, E. J.) vol. 546 1–20 (Academic Press, 2014).

11. Demichev, V., Messner, C. B., Vernardis, S. I., Lilley, K. S. & Ralser, M. DIANN: neural networks and interference correction enable deep proteome coverage in high throughput. *Nat. Methods* **17**, 41–44 (2020).

12. Tyanova, S. *et al.* The Perseus computational platform for comprehensive analysis of (prote)omics data. *Nat Methods* **13**, 731–40 (2016)

13.. Abramson, J. *et al.* Accurate structure prediction of biomolecular interactions with AlphaFold 3. *Nature* **630**, 493–500 (2024).
