# Supplementary material for: CUL4A-DDB1-DCAF10 is an N-recognin for N-terminally acetylated Src kinases
Source: Nat Commun. 2026 Jan 3;17:132. doi: 10.1038/s41467-025-68074-9 (PMC12775124; doi:10.1038/s41467-025-68074-9)
Supplement: Supplementary file 2 — Description of Additional Supplementary Files [file 41467_2025_68074_MOESM2_ESM.pdf]

Description of Additional Supplementary Files

File Name: **Supplementary Data 1**

Description: Mass spectrometry label-free quantification data and processed statistical results used to generate the volcano plots in **Figure 1.**

File Name: **Supplementary Data 2**

Description: Mass spectrometry label-free quantification data and processed statistical results used to generate the volcano plots in **Supplementary Figure 1.**

File Name: **Supplementary Data 3**

Description: Mass spectrometry label-free quantification data and processed statistical results used to generate the volcano plots in **Figure 2**.

File Name: **Supplementary Data 4**

Description: Mass spectrometry label-free quantification data used for DCAF10 quantification in control and siRNA treated cells in **Figure 4.**

File Name**: Supplementary Data 5**

Description: Mass spectrometry label-free quantification data used for ZYG11B quantification in control and siRNA treated cells in **Figure 4.**

File Name: **Supplementary Data 6**

Description: Mass spectrometry label-free quantification data used to confirm Lyn KO and compare proteomes of KO clones and parental cells in **Supplementary Figure 7**.

File Name: **Supplementary Data 7**

Description: Mass spectrometry label-free quantification data and processed statistical results used to generate the volcano plots in **Supplementary Figure 7**.

File Name: **Supplementary Data 8**

Description: Mass spectrometry label-free quantification data and processed statistical results used to generate the volcano plots in **Figure 6**.
