# Supplementary material for: CUL4A-DDB1-DCAF10 is an N-recognin for N-terminally acetylated Src kinases
Source: Nat Commun. 2026 Jan 3;17:132. doi: 10.1038/s41467-025-68074-9 (PMC12775124; doi:10.1038/s41467-025-68074-9)
Supplement: Supplementary file 11 — Reporting Summary [file 41467_2025_68074_MOESM11_ESM.pdf]

nature portfolio

Corresponding author(s): Tanja Bange

Last updated by author(s): Dec 15, 2025

Reporting Summary

Nature Portfolio wishes to improve the reproducibility of the work that we publish. This form provides structure for consistency and transparency in reporting. For further information on Nature Portfolio policies, see our Editorial Policies and the Editorial Policy Checklist.

Statistics

For all statistical analyses, confirm that the following items are present in the figure legend, table legend, main text, or Methods section.

n/a	Confirmed	
☐	☒	The exact sample size (n) for each experimental group/condition, given as a discrete number and unit of measurement
☐	☒	A statement on whether measurements were taken from distinct samples or whether the same sample was measured repeatedly
☐	☒	The statistical test(s) used AND whether they are one- or two-sided<br>*Only common tests should be described solely by name; describe more complex techniques in the Methods section.*
☒	☐	A description of all covariates tested
☒	☐	A description of any assumptions or corrections, such as tests of normality and adjustment for multiple comparisons
☐	☒	A full description of the statistical parameters including central tendency (e.g. means) or other basic estimates (e.g. regression coefficient) AND variation (e.g. standard deviation) or associated estimates of uncertainty (e.g. confidence intervals)
☐	☒	For null hypothesis testing, the test statistic (e.g. F, t, r) with confidence intervals, effect sizes, degrees of freedom and P value noted<br>*Give P values as exact values whenever suitable.*
☒	☐	For Bayesian analysis, information on the choice of priors and Markov chain Monte Carlo settings
☒	☐	For hierarchical and complex designs, identification of the appropriate level for tests and full reporting of outcomes
☒	☐	Estimates of effect sizes (e.g. Cohen's d, Pearson's r), indicating how they were calculated

Our web collection on statistics for biologists contains articles on many of the points above.

Software and code

Policy information about availability of computer code

Data collection	In this study, no custom-made software or codes/mathematical algorithms were generated by the authors. For data collection commercial softwares were used: Image Lab Software (version 6.0.1, BIO-RAD, Western blot imaging), Xcalibur (version 4.6, Thermo Scientific, MS data acquisition), SparkControl (version 3.2, Tecan Spark Multimode Microplate Reader, Tecan, N-terminal acetylation assays). Software programs used are summarized in Supplementary Table 10.
Data analysis	In this study, no custom-made software or codes/mathematical algorithms were generated by the authors. For data analysis commercial or freely available software programs were used: Perseus (version 1.6.50, 2.0.11, https://maxquant.net/perseus/), DIA-NN (1.8.2. beta 22 and 27, https://github.com/vdemichev/DiaNN), ImageJ (version 2.0.0-rc-69/1.52p, National Institutes of Health), Resovle3D softWoRx-Aquire (version 7.2.0; Release RC4), PyMOL (version 3.0, Schrödinger, LLC), Rstudio (version 2023.06.0, Prosit Software, PBC), GraphPad Prism (version 8.4.3, GraphPad Software, LLC), SnapGene (version 7.1.2, GSL Biotech LLC). BioRender for schematics. Structural predictions were carried out with AlphaFold 3.0.0. Software used is summarized in Supplementary Table 10.

For manuscripts utilizing custom algorithms or software that are central to the research but not yet described in published literature, software must be made available to editors and reviewers. We strongly encourage code deposition in a community repository (e.g. GitHub). See the Nature Portfolio guidelines for submitting code & software for further information.

Data

Policy information about availability of data

All manuscripts must include a data availability statement. This statement should provide the following information, where applicable:

- Accession codes, unique identifiers, or web links for publicly available datasets
- A description of any restrictions on data availability
- For clinical datasets or third party data, please ensure that the statement adheres to our policy

The datasets generated and/or analysed during this study are publicly available. Mass spectrometry raw data, search engine outputs and experimental design files have been deposited in the PRIDE/ProteomeXchange repository under accession PXD061095. The Source Data file, including all uncropped Western blots (224 files) and the corresponding quantification tables, is available on Figshare (DOI: 10.6084/m9.figshare.30450536). Newly generated cell lines, plasmids and other reagents are available from the corresponding author upon request.

Research involving human participants, their data, or biological material

Policy information about studies with human participants or human data. See also policy information about sex, gender (identity/presentation), and sexual orientation and race, ethnicity and racism.

Reporting on sex and gender	Sex and gender are not relevant in this study. We did not have human participants or material from human participants.
Reporting on race, ethnicity, or other socially relevant groupings	Race, ethinicity, or socially relevant groupings are not relevant for this study.
Population characteristics	Population characteristics are not relevant for this study.
Recruitment	Recruitment is not relevant for this study.
Ethics oversight	Ethics oversight is not relevant for this study.

Note that full information on the approval of the study protocol must also be provided in the manuscript.

Field-specific reporting

Please select the one below that is the best fit for your research. If you are not sure, read the appropriate sections before making your selection.

☒ Life sciences ☐ Behavioural & social sciences ☐ Ecological, evolutionary & environmental sciences

For a reference copy of the document with all sections, see nature.com/documents/nr-reporting-summary-flat.pdf

Life sciences study design

All studies must disclose on these points even when the disclosure is negative.

Sample size	No statistical sample size calculation was performed. Sample sizes followed established standards for biochemistry, proteomics, and cell biology. Mass spectrometry experiments were performed in biological triplicates, in vitro enzymatic assays in 3–4 independent replicates, and cell culture experiments in 3–6 biological replicates. These replicate numbers are standard in the field and were sufficient to ensure reproducible detection of experimental effects.
Data exclusions	No data were excluded from the analyses unless predefined quality-control filtering inherent to standard proteomics workflows was applied (e.g., removal of proteins not detected in 3/3 replicates before imputation). No experimental samples were removed.
Replication	All key findings were reproduced across multiple independent biological replicates. MS experiments were repeated in triplicate, Western blot quantifications in triplicate, enzymatic assays in 3–4 replicates, and cell-based experiments in 3–6 biological replicates. All attempts at replication were successful.
Randomization	Randomization was not applicable. Experimental groups were defined by fixed biochemical or genetic conditions (e.g., peptide identity, siRNA treatment, CRISPR knockout, doxycycline induction) and all samples were processed in parallel under identical conditions. No covariates required balancing..
Blinding	Blinding was not relevant to the study. Data acquisition was performed using automated systems (LC–MS, fluorescence measurements, imaging), and data analysis was carried out using software-based pipelines (DIA-NN, Perseus, ImageJ). Group assignment was defined by experimental conditions and not operator judgment.

Reporting for specific materials, systems and methods

We require information from authors about some types of materials, experimental systems and methods used in many studies. Here, indicate whether each material, system or method listed is relevant to your study. If you are not sure if a list item applies to your research, read the appropriate section before selecting a response.

Materials & experimental systems

n/a	Involved in the study
	☒ Antibodies
	☒ Eukaryotic cell lines
☒	Palaeontology and archaeology
☒	Animals and other organisms
☒	Clinical data
☒	Dual use research of concern
☒	Plants

Methods

n/a	Involved in the study
☒	ChIP-seq
☒	Flow cytometry
☒	MRI-based neuroimaging

Antibodies

Antibodies used

A list of antibodies with detailed information is in Supplementary Method Table 6.
anti-CUL4A Rabbit pAb, Cell Signaling, #2699; dilution: 1:1000
anti-DCAF10/ WDR32 Rabbit pAb, Thermo Fisher Scientific, #PA5-24133; dilution: 1:1000
anti-DDB1 (D4C8) Rabbit mAb, Cell Signalling, #6998; dilution: 1:1000
anti-FLAG (DYKDDDK) Mouse mAb, Cell Signaling , #8146S; dilution: 1:5000
anti-Fyn Rabbit pAb, Cell Signaling , #4023S; dilution: 1:1000
anti-GFP Rabbit pAb, n-house, AG Musacchio; dilution: 1:1000
anti-GFP Mouse mAb, Roche, #11814460001; dilution: 1:10000
anti-GST Mouse mAb, Merck Millipore, #71097; dilution: 1:1000
anti-Lyn (C13F9) Rabbit mAb, Cell Signaling, #2796S: dilution: 1:1000
anti-mouse IgG HRP linked antibody, Cell Signaling, #CST 7076S; dilution: 1:10000
anti-NMT1 Rabbit pAb, abcam, #AB186123; dilution: 1:1000
anti-NMT2 Rabbit pAb, abcam, #AB230028; dilution: 1:1000
anti-rabbit IgG HRP-linked Antibody, Cell Signaling, #7074S; dilution: 1:10000
anti-Src (36D10) Rabbit mAb, Cell Signaling, #2109S; dilution: 1:1000
anti-Streptavidin Rabbit pAb, Rockland immunochemicals, #100-4195; dilution: 1:10000
anti-THOC7 Rabbit pAb, abcam, #ab155218; dilution 1:500
anti-Ubiquitin-HRP (P4D1) Mouse mAb, Cell Signaling, #14049; dilution: 1:1000
anti-Vinculin Mouse mAb, Sigma, #V9131; dilution: 1:1000
anti-ZER1 Rabbit pAb, Proteintech, #16647-1-AP: dilution: 1:1000
anti-ZYG11B Rabbit pAb, antibodies-online.com, #ABIN4916715; dilution: 1:500
VeriBlot Detection Reagent (for IP samples), abcam, #ab131366; dilution: 1:500

Validation

All commercial primary antibodies used in this study were validated by the respective suppliers (see product information sheets) and have been widely used in published studies. Each antibody was further validated in-house by confirming detection of a single band at the expected molecular weight in SDS–PAGE/Western blot experiments, and in several cases by loss of signal following siRNA-mediated depletion of the target protein. The in-house–generated GFP antibody has been validated previously (e.g., Alex et al., eLife 2019) and has been routinely used in our laboratory.

Eukaryotic cell lines

Policy information about cell lines and Sex and Gender in Research

Cell line source(s)

We provide a list of all cell lines used with reference or generated in this study with details in Supplementary Method Table 2 and the methods section. Cell lines lare available from the corresponding author.
DLD-1 Flp-In™ T-REx™, gift from A. Musacchio already used in publications
DLD-1 Flp-In™ T-REx™ Lyn KO, generated in this study
DLD-1 KO Lyn Flp-In™ T-REx™ LynG2A-GFP, generated in this study
DLD-1 KO Lyn Flp-In™ T-REx™ LynG2P-GFP, generated in this study
DLD-1 KO Lyn Flp-In™ T-REx™ LynWT-GFP, generated in this study
Hela Flp-In™ T-REx™, a gift from S. Taylor (University of Manchester)
RPE-1 Flp-In™ T-REx™, a gift from AG Musacchio

Authentication

All cell lines used in this study (HeLa, RPE-1, DLD-1 Flp-In T-REx, and the DLD-1 Lyn KO–derived Lyn-GFP lines) were regularly tested and confirmed to be mycoplasma-free. The parental HeLa, RPE-1, and DLD-1 Flp-In T-REx lines were obtained from established, authenticated laboratory stocks as listed in Supplementary Table 2. Newly generated DLD-1 Lyn KO and Lyn-GFP variant lines were validated by genomic sequencing, Western blotting, and mass spectrometry (see Methods).

Mycoplasma contamination

All cell lines were tested negative against Mycoplasma contamination.

Commonly misidentified lines
(See ICLAC register)

No misidentification issues are known for any cell line used.

Plants

Seed stocks	We did not use any plant material in this study.
Novel plant genotypes	We did not use any plant material in this study.
Authentication	We did not use any plant material in this study.
